# Venom Before Specialisation? A Layered Assessment of Early Venom Evolution in Lizards and Snakes

**DOI:** 10.3390/toxins18100409

**Published:** 2026-09-23

**Authors:** Bryan G. Fry

**Affiliations:** Adaptive Biotoxicology Lab, School of the Environment, University of Queensland, St Lucia, QLD 4072, Australia; bgfry@uq.edu.au

**Keywords:** venom system, Toxicofera, Anguimorpha, Serpentes, ancestral-state reconstruction, character coding, gland evolution

## Abstract

Debate over the origin of squamate venom is frequently distorted by treating molecular availability, toxin recruitment and secretion, functional venom, and delivery-system elaboration as a single evolutionary event. This review separates these transitions and reassesses early venom evolution in anguimorph lizards and snakes using anatomical, molecular, proteomic, functional, ecological, and phylogenetic evidence. The species-level ancestral-state reconstructions of a recent Wüster, Modahl, and Arbuckle manuscript are internally robust to the four coding schemes tested and accommodate uncertain tips and reversible venom loss. Their inference nevertheless remains conditional on a binary definition of observed functional venom, a single hidden precursor category, a homogeneous transition process, and non-caenophidian snake states that were not varied in the relevant sensitivity analyses. While they coded two origins of venom within Anguimorpha, gland anatomy and derived oral products make an early functional origin followed by retention, reduction, loss, and parallel elaboration a strongly supported alternate hypothesis. However, as critical bridge taxa lack direct evidence of natural delivery, realistic dose, or secretion-specific fitness benefit, resolution of the history remains unresolved. For *Heloderma*, field studies of *H. suspectum* and *H. horridum*, together with painful, prolonged defensive bites, support a defensive role, although its primacy (to the exclusion of an additional predatory function) as advocated by them is sensitive to observational bias. Neurotoxic, cardiovascular, and coagulotoxic activities make a predatory contribution supported, leaving the original selective driver unresolved. Within Serpentes, their placement of venom at the base of the Caenophidia is challenged by distributed maxillary, mandibular, and rictal glands and derived oral products in non-caenophidian taxa, making an origin before Caenophidia a viable alternative hypothesis. Derived mucous systems in boas and pythons and specialised Scolecophidia cannot alone polarise the ancestral snake state. Current evidence is therefore insufficient to distinguish one shared functional origin from two functionalisations of a homologous ancestral oral secretory system. No alternative comparative reconstruction is performed; the review shows only that earlier origins remain viable and categorical absence coding is insufficiently secure for several poorly investigated taxa. Resolution requires matched gland cell biology, orthology- and synteny-resolved genomics, quantitative secretion proteomics, realistic functional assays, secretion-specific ecological tests, broader Iguania and non-caenophidian snake sampling, and ancestral-state models that represent graded evidence, both false-negative and false-positive classification, reduction, loss, and phylogenetic uncertainty.

## 1. Introduction: What Counts as an Origin? The Conceptual and Evidentiary Framework for Evaluating Early Venom Origins in Squamate Reptiles

### 1.1. Opening Problem

Arguments about the origin of venom in lizards and snakes often appear to concern a single historical event. In practice, they frequently combine several events that need not have coincided: the availability of a physiological protein in oral tissue; its recruitment and secretion as a toxin; the establishment of a fitness-relevant venom function; the anatomical specialisation of the gland; the modification of teeth; and the evolution of forceful or high-pressure delivery. Treating these as interchangeable produces a recurrent category error. A lineage may possess a secretion that contributes to prey subjugation or defence without possessing a discrete, encapsulated venom gland, a dedicated compressor muscle, a grooved fang, or a pressure-injection mechanism. Conversely, specialised teeth or glandular anatomy cannot, by themselves, establish that the secretion was toxic, ecologically deployed, or selected for venom function. The first requirement of any origins analysis is therefore to specify which trait is claimed to have originated.

This distinction follows from a functional definition of venom. Venom is best treated as a secretion produced by specialised cells and delivered through an inflicted wound, where it perturbs the physiology of another organism in a way that contributes to a fitness-relevant interaction [1]. The interaction can involve predation, defence, competition, intraspecific conflict, or another demonstrable biological role; it need not involve a syringe-like apparatus, and the secretion need not be indispensable to every feeding event. The wider literature has converged on active wound-mediated delivery as a central distinction while continuing to debate how much evidence is needed to infer selected function [1,2,3,4,5,6,7].

On this definition, a modest serous or seromucous secretion can be selectable long before the appearance of a visually conspicuous apparatus. A bite already creates a wound. If oral proteins entering that wound incrementally prolong prey contact, reduce escape probability, shorten immobilisation time, lessen retaliatory injury, or deter a predator, then selection can act on secretion composition, expression, and volume before it acts on encapsulation, duct geometry, dental grooves, or compressor muscles. Early venom systems may consequently be anatomically diffuse, behaviourally conditional, quantitatively weak, and easy to miss when modern front-fanged snakes are treated as the template for recognition. This is not an argument that every bioactive oral secretion is venom. It is an argument that the functional threshold and the architectural threshold are different thresholds. Comparative morphology and modern analyses of evolutionary novelty likewise support separating the first functional secretion from the later optimisation of its delivery apparatus [8,9].

The modern debate was sharpened by the proposal that a venom system arose early in the common ancestry of toxicoferan squamates [10]. That proposal combined the distribution of protein-secreting oral glands, phylogenies of toxin-related sequences, and functional assays into an inference of a shared early system [10,11]. Subsequent syntheses explicitly described a plesiomorphic oral secretory condition followed by divergent elaboration, reduction, and loss [11,12,13].

The immediate stimulus for revisiting what ‘origin’ means is the recent review by Wüster, Modahl, and Arbuckle [14]. Its hidden-state ancestral reconstructions infer venom at the root of Colubroidea, possibly following an immediately antecedent precursor, but infer a separate precursor within Anguimorpha and multiple origins of functional venom within anguimorph lizards under the principal coding schemes. The number and placement of those anguimorph origins vary according to whether varanoids and other anguimorphs are scored as venomous. This is an important advance over a direct two-state reconstruction because it acknowledges that a nonvenomous precursor can precede functional venom. At the same time, the results are conditional on the biological definition assigned to the tips and on whether incompletely studied lineages enter the analysis as absent or unknown.

That conditionality is central rather than incidental. The published reconstruction by Wüster, Modahl, and Arbuckle is shown as a simplified diagram in which ‘early-diverging snakes’ appear as a broad visual branch, but the underlying analysis uses 4162 species-level tips across all lizards and snakes, including early-diverging species, and treats taxa with substantial uncertainty as unknown [14]. Analytical aggregation is therefore not a defect of that study. The more specific limitation is that sampled non-caenophidian snake tips relevant to an earlier snake origin (such as *Cylindrophis* and *Eryx* species) remain coded nonvenomous across the four schemes, whereas the sensitivity analyses chiefly alter debated anguimorphs and a derived Caenophidian snake group. Within Anguimorpha, the exact position of the precursor and the number of subsequent functional gains change, but the broader inference of a single Anguimorpha precursor followed by separate functional origins in the clades treated as venomous remains stable. This conditional robustness makes it necessary to test biologically explicit alternatives rather than infer them from the simplified figure.

Consequently, the present manuscript adopts a layered question. It first asks what molecular, anatomical, secretory, functional, and ecological evidence exists within lizards and within snakes. Only then does it ask whether those layers support a shared origin of functional venom, a shared pre-venom substrate followed by separate acquisitions of function, two wholly separate origins, or a conclusion of present data deficiency. This ordering prevents a derived fang or compressor muscle from being mistaken for the beginning of venom itself.

This article is a hypothesis-driven narrative synthesis, rather than a systematic review. It prioritises evidence that bears directly on the distinction among molecular availability, toxin secretion, functional venom, reduction or loss, and delivery-system elaboration; gives particular weight to phylogenetically informative taxa and convergence among anatomy, molecular identity, proteomics, realistic functional assays, ecology, and comparative modelling; and considers rigorous negative evidence alongside positive evidence. It does not present a new species-level recoding or ancestral-state analysis and therefore does not estimate the relative support of one historical model over another. Proposed alternative codings and models are framed as targets for future, evidence-audited comparative tests, not as new quantitative results.

### 1.2. Four Events That Must Be Separated

#### 1.2.1. Molecular Availability

The earliest and weakest evidentiary level is molecular availability: a physiological gene or protein family is present and expressed in oral tissue. This matters because evolutionary novelty normally modifies pre-existing biology. Many protein families later exploited in venoms have ancient roles in haemostasis, digestion, immunity, inflammation, cell signalling, or tissue remodelling [1,15], and oral secretory epithelia provide a plausible context in which those proteins can contact another organism. Yet the presence of a homologue, even in an oral gland, is not equivalent to toxin recruitment. It may represent low-level transcription, a broadly expressed housekeeping function, a salivary or antimicrobial role, or ancestral regulatory accessibility without toxicity [16,17].

Comparative transcriptomics made this limitation unavoidable. Venom-gene homologues in python tissues are not confined to oral glands, and several proposed toxin families show broad physiological expression across reptiles [18]. Those findings weakened the practice of treating sequence similarity or a single transcript as sufficient evidence of venom, while leaving open the possibility that oral expression supplies material for later restriction, duplication, regulatory intensification, or neofunctionalisation. However, it must also be noted that in this study the well-characterised toxin types 3FTx and lectins were dominantly expressed in the oral glands relative to other tissues [18].

Regulatory similarity is likewise evidence of predisposition, not necessarily of venom function [19]. A conserved salivary gene-regulatory network may make repeated oral recruitment more probable across amniotes, or it may preserve a homologous platform from which a shared early toxic secretion evolved. Either interpretation concerns the accessibility of an evolutionary route. It does not, without secretion and function data, determine when the route was first used as venom.

#### 1.2.2. Toxin Recruitment and Secretion

A stronger event occurs when a physiological precursor gives rise to an isoform or paralogue that is preferentially deployed by oral secretory cells, translated, processed, and released. ‘Recruitment’ should therefore describe a change in biological deployment, not merely membership in a protein family already known from venom. Evidence is strongest when gene phylogeny identifies the relationship between toxin and non-toxin homologues; synteny and genomic context distinguish orthology, duplication, and loss; sequence analysis identifies structural innovation or altered selection; and regulatory data show oral enrichment or restriction [1,15]. Because duplication, restriction, co-option, and changes in dosage can all contribute, no single universal molecular route should be assumed.

Gene-tree analyses have shown both recruitment and reversal, with toxin status gained and lost dynamically within protein families [20]. This finding is important in two directions: a shared toxin-like sequence need not prove a single functional origin; and the absence of a toxin role in a living lineage need not indicate that its ancestor lacked one.

Transcription is still only potential production. A candidate toxin must be shown to enter the secretion. Quantitative proteomics, peptide-level validation, and, where feasible, intact-mass or targeted confirmation are needed to demonstrate translation and release, to distinguish highly abundant venom components from trace proteins, and to guard against contamination from blood, muscle, epithelia, or adjacent glands. Multi-tissue work in *Bothrops jararaca* illustrates why abundance and tissue context are decisive: some venom-related transcripts occur outside the venom gland, while the dominant toxin complement is strongly enriched within it [21]. Proteomic inventories are therefore not merely descriptive additions to transcriptomes; they establish which candidate molecules are actually packaged into the material delivered during a bite [22]. The methodological development of venomics reinforces this requirement: integrated proteomic workflows can distinguish mature secreted products, proteoforms, processing, and relative abundance from a transcript list that records only production potential [23].

#### 1.2.3. Functional Venom

Functional venom is the point at which the secretion becomes an organismal trait rather than a molecular possibility. The secretion must be delivered through a wound and must alter recipient physiology in a way that contributes to a selected interaction. Demonstrating that contribution does not require showing that the animal is incapable of capturing prey without venom. Evolution can favour a chemically induced incremental reduction in handling time, prey escape, energetic expenditure, or injury risk. Nor does the target have to die: pain, hypotension, coagulotoxicity, neurotoxicity, tissue disruption, or sensory disturbance may be sufficient if they improve performance in the relevant context.

Biochemical activity alone is not conclusive. Almost any concentrated biological secretion can produce an effect in a permissive assay, and injection into an artificial model bypasses the dose, route, and tissue exposure achieved by a natural bite. Functional evidence becomes persuasive when effects occur at secretion-relevant concentrations, by a realistic route, and in physiologically relevant prey or predator targets. Prey-specific activity and correlations between diet and venom performance demonstrate why assays restricted to laboratory mice or generic plasma can mischaracterise ecological function [24,25,26,27,28,29,30,31,32,33,34,35,36,37,38,39,40,41,42,43,44,45,46,47].

#### 1.2.4. Delivery-System Elaboration

Delivery-system elaboration is the fourth event. Protein-secreting fields can become enlarged, segregated from mucous compartments, encapsulated, and connected to ducts; surrounding muscles can become compressors; teeth can become enlarged, ridged, grooved, canaliculated, hinged, or repositioned; and biting behaviour can increase the duration or pressure of delivery. Each modification may increase dose, speed, precision, or reliability. None is required for the first weak venom function, and none should be allowed to define that function retrospectively.

The anatomy itself supports a distinction between shared substrates and repeated elaboration. Ophidian oral glands form a diverse set of homologous and serially related secretory structures whose names have sometimes carried unwarranted functional assumptions [48,49]. Within Anguimorpha lizards, basal mixed seromucous glands and derived encapsulated protein-secreting glands document extensive structural divergence, including independent segregation and enlargement of protein-secreting compartments [10,50,51].

Snake delivery systems likewise show repeated modifications of tooth position, groove depth, fang mobility, duct association, and gland compression [48]. Developmental evidence indicates a shared posterior dental developmental field for snake fangs, while the architectures of rear-fanged, elapid, viperid, and atractaspid venom systems have followed distinct evolutionary trajectories [48,49,52,53].

The central interpretive rule follows: independent elaborations of delivery architecture do not, by themselves, demonstrate independent origins of the underlying venom function. Architecture may diagnose later optimisation of a system that was already functional, or it may arise after a previously nonvenomous precursor crosses the functional threshold. Determining which history occurred requires evidence from the other three levels. The four evolutionary stages, the separate functional and architectural thresholds, and the possibility of reduction or loss are summarised in Figure 1.

### 1.3. Evidence Hierarchy and Coding Rules

The evidence should be treated as a linked hierarchy, not as a single decisive test. Anatomy establishes the tissue and route through which a secretion could be produced and delivered. Molecular analyses identify candidate derived toxins and their evolutionary relationships. Proteomics of expelled material can establish secretion, whereas functional assays establish physiological actions. Ecology establishes whether that action changes an outcome on which selection can operate. The farther a claim moves from molecular availability towards functional venom, the more it should rest on convergence among these evidence classes. A missing layer should lower confidence without automatically being treated as evidence of absence. Conversely, a positive anatomical, molecular, or pharmacological observation should not be promoted to functional venom when natural secretion, realistic delivery, or fitness relevance remains untested. Rigorous negative evidence should reduce support for a positive state. Probabilistic coding should therefore preserve both directions of evidence and accommodate false-negative and false-positive classification.

#### 1.3.1. Anatomy

Anatomical investigation should begin with the complete oral secretory field rather than with a search for a single structure resembling a viper venom gland. Histology and three-dimensional anatomy should identify serous or seromucous protein-secreting compartments, storage lumina, ducts and duct openings, vascular and muscular relationships, and the spatial relation between secretion release and the teeth or wounds produced during biting [48,49,51,54]. Secretory polarity and evidence of stored material strengthen the inference that proteins are exported into the oral secretion. However, a serous gland is evidence of secretory capacity, not automatically of venom; its products and their use still require testing. This whole-field approach is grounded in classical comparative descriptions of ophidian cephalic and salivary glands and in later work showing both the internal specialisation of front-fanged venom glands and substantial functional variation among other oral glands [48,49,55,56,57,58].

#### 1.3.2. Molecular Identity

Molecular identity should be assigned to specific orthologues, paralogues, or isoforms rather than to broad family names. Gene phylogeny, synteny, exon structure, domain architecture, duplication history, sequence innovation, selection, expression level, cellular localisation, and regulatory context should be used together. This matters because the nearest physiological homologue of a venom protein can be expressed in many tissues, while a toxin paralogue may be narrowly enriched in secretory cells [1,15]. It also prevents circular reasoning in which a transcript is called a toxin because it resembles a database toxin and the species is then called venomous because it expresses the transcript.

Genomic studies illustrate why this resolution matters: novel toxin families can arise through identifiable duplication and sequence innovation, deeply divergent toxin-gene haplotypes can encode alternative venom phenotypes within a species, and apparently simple venoms can rest on unexpectedly complex genomic architectures [59,60,61].

#### 1.3.3. Secretion

Secretion should be demonstrated directly. Ideally, the sampled material should be collected from the relevant oral region under conditions that minimise tissue damage and blood contamination, with biological replication and negative controls. Proteomic identification should account for shared peptides among closely related paralogues and should report abundance rather than relying only on presence. Stimulation history is relevant because secretory systems can change dramatically after depletion; failure to detect a component in a quiescent or poorly sampled gland is not necessarily a true negative. Where feasible, spatial proteomics, immunohistochemistry, or targeted mass spectrometry can connect a candidate toxin to the cell type and compartment that releases it. Experimental systems now permit the secretory process itself to be interrogated: venom-gland organoids reproduce toxin production and release, while spatially resolved analyses show that toxin output is heterogeneous within a gland and constrained by secretory physiology [62,63].

#### 1.3.4. Physiological Function

Function should be tested using the whole secretion and, where attribution is needed, purified or recombinant components. Dose should be scaled to plausible yield and delivery, and assays should distinguish general cytotoxicity from targeted physiological disruption. Relevant endpoints include neuromuscular transmission, coagulation, platelet function, vascular tone, pain pathways, tissue integrity, and prey-specific toxicity, but assay choice must follow the proposed ecological role. A positive effect at an unrealistically high concentration is evidence of biochemical capability, not yet of venom function.

#### 1.3.5. Ecological Evidence and the Counterfactual Problem

Natural-history observations remain indispensable for identifying which interactions occur, which recipients are relevant, whether biting is prolonged, whether jaw movements are consistent with secretion transfer, and which outcomes merit measurement. They can make a proposed predatory, defensive, competitive, or other role more or less plausible. They do not, however, isolate the effect of venom. In a free-ranging encounter, apparent prey impairment follows a composite exposure that may include puncture, laceration, crushing, restraint, constriction, exhaustion, shock, stress, and toxin delivery. Without a secretion-specific counterfactual, a prey item that slows after being bitten cannot reveal which component caused the change. Natural history therefore supplies ecological context and testable hypotheses, but observation alone is not a reproducible diagnostic test of venom function [1,2,3,4,5,6,7].

A causal demonstration must alter exposure to the secretion while holding the remaining interaction as constant as possible. Depending on the taxon, welfare constraints, and proposed mechanism, designs could use validated temporary duct occlusion, controlled gland depletion followed by quantified recovery, recipient-side neutralisation of defined components, or matched application of collected secretion to a standardised bite-like wound, each paired with appropriate sham controls. No intervention is universally valid: it must be shown not to alter motivation, bite force, tooth penetration, bite duration, handling behaviour, prey condition, or recipient physiology independently of venom. Trials should use ecologically relevant recipients and plausible doses, and should pre-specify outcomes such as escape probability, time to incapacitation, handling duration, retaliatory injury, energetic cost, predator withdrawal, or learned avoidance. Field observation identifies the behaviour and context to reproduce; the manipulation supplies the missing causal attribution.

No universal magnitude of ecological effect can define the boundary between a bioactive oral secretion and venom. Fitness relevance is an evolutionary criterion, not a requirement that an observer witness rapid paralysis or prey collapse. A small probabilistic reduction in escape, handling time, injury, or predator persistence can be selectable. Secretion-specific ecological experiments provide the strongest evidence of present venom function when a plausibly delivered dose causes a reproducible recipient-directed physiological effect and a validated contrast improves a pre-specified performance outcome. They are not, however, a universal minimum that every otherwise well-supported venom must satisfy; converging anatomical, molecular, proteomic, physiological, behavioural, and clinical evidence can support functional classification when such an experiment is impractical or unethical. Historically selected function must then be inferred from the conjunction of anatomy, derived molecular characters, tissue-specific production, secretion, realistic activity, comparative distribution, and present performance because the ancestral selective event cannot itself be observed. Studies should define the smallest biologically important effect before testing and report effect sizes, confidence intervals, delivered-dose variation, and individual heterogeneity. An undefined threshold such as ‘obvious prey impairment’ will systematically favour high-output systems with fast, visible effects.

Negative ecological results should be evaluated by the same graded standard. A non-significant result, or failure to witness a conspicuous response in the field, provides limited evidence against function, particularly for low-output, weakly acting, conditionally deployed, or prey-specific secretions. By contrast, a rigorous negative study that tests realistic recipients, doses, behaviours, and life stages with enough precision to exclude a pre-specified biologically meaningful benefit should reduce support for present venom function and may support absence within those clearly defined conditions; it does not establish unconditional absence beyond them. Wüster, Modahl, and Arbuckle are correct that natural history is crucial for understanding venom ecology, but their proposal that ecological and natural-history data are exactly what is needed to resolve controversial taxa does not specify a secretion-specific counterfactual or criteria for accepting and rejecting the venom hypothesis [14]. Without such rules, incomplete investigation should not be converted to a nonvenomous tip state, while informative negative results should not automatically be converted to unknown; each should be coded according to its direction, quality, scope, and precision.

#### 1.3.6. Tip States

These evidentiary distinctions must be carried into phylogenetic coding. ‘Absent’, as used by Wüster, Modahl, and Arbuckle [14], should be supported by an appropriate negative investigation for the state in question, just as a positive functional-venom code should be supported by evidence relevant to natural secretion, wound delivery, realistic dose, physiological effect, and fitness relevance. A taxon can be demonstrably without a high-pressure fang system while remaining unknown for functional venom, positive for a serous oral gland, and uncertain for toxin secretion. Unsampled taxa should be coded as unknown rather than nonvenomous. Where evidence is graded, tip likelihoods or probabilistic states should represent the probability of both false-negative and false-positive classification. Rigorous negative results should lower the probability of presence and, when sufficient for the defined state and conditions, may support an absent code; an unknown state is appropriate when the available evidence is genuinely uninformative or inadequate. In short, absence of evidence is not evidence of absence, but evidence of absence should be retained.

Evidence quality is state-specific in both directions. An appropriate negative investigation must address the state actually being coded. For negative anatomical coding, this requires adequate histology of the relevant oral regions. For a negative secretion code, it requires sensitive proteomics of properly collected material. For a negative physiological-function code, it requires plausible doses, routes, and targets capable of detecting the proposed effect. For a negative ecological-function code, it requires a validated secretion-specific counterfactual, realistic recipients and doses, and sufficient precision to exclude a pre-specified biologically meaningful benefit. Uncontrolled natural-history observations can document behaviour and possible delivery, but they cannot establish secretion-specific causation, and failure to observe an obvious effect cannot establish absence. Failure at one level does not negate evidence at another. This coding discipline is especially important in early-diverging snakes and poorly sampled lizards, where the absence of a familiar apparatus or a conspicuous field effect has often been allowed to stand in for the absence of a functional secretion. These standards apply symmetrically: well-designed negative studies should reduce support for presence, whereas weak positive or negative studies should carry correspondingly little weight.

Phylogenetic depth must not be conflated with morphological primitiveness. The predominantly mucous labial glands of boas and pythons are plausibly a derived accompaniment to constriction and the lubrication of large prey [64], whereas Scolecophidia have highly modified skulls, restricted gape, and specialised fossorial feeding on ants, termites, and their eggs, larvae, and pupae [49]. A negative venom assessment in either group therefore describes an extant, ecologically transformed condition; it cannot, by itself, establish absence in the snake ancestor.

### 1.4. Why Binary Ancestral-State Reconstructions Are Vulnerable

A binary venom/no-venom character as per Wüster, Modahl, and Arbuckle [14] compresses a clinal sequence of biologically distinct states into a single threshold. Molecular availability, oral enrichment, secretion, recipient activity, ecological benefit, gland encapsulation, and dental specialisation may evolve at different times and can be lost or reduced independently. When all are represented by one bit, disagreements over a taxon may reflect different threshold definitions rather than different observations. One investigator may code a lineage positive because its secretion contains active proteins delivered by biting, while another may code it negative because the teeth are unspecialised or ecological benefit has not been quantified. The resulting ancestral-state contrast appears numerical but is partly semantic.

Hidden-state models are a constructive response because they allow an observed nonvenomous condition to contain an unobserved precursor and permit historical questions that direct binary models cannot express. The precursor framework used by Wüster, Modahl, and Arbuckle [14] has a clear comparative-methods foundation [65]. More generally, hidden Markov models can accommodate unobserved rate classes and reduce the unrealistic assumption that one transition process applies across an entire tree [66].

A single hidden ‘precursor’ state such as that used by Wüster, Modahl, and Arbuckle [14] may nevertheless remain too coarse for the venom problem. A serous gland without toxin enrichment, oral enrichment without demonstrated translation, secretion without realistic activity, and activity without established ecological benefit are not biologically equivalent. Collapsing all of them into one hidden state assumes that they share the same transition structure and proximity to functional venom. It can also conceal alternative routes: ecological function might arise before gross gland specialisation in one lineage, while anatomical focusing precedes strong toxicity in another. A more realistic analysis may require several ordered or partially ordered states, while allowing some transitions to be bypassed and others to reverse. The wider comparative-methods literature provides the same caution. Ancestral-state estimates can be sensitive to model structure and character coding, while structured and generalised hidden Markov approaches offer ways to represent multiple latent biological conditions rather than forcing them into one precursor category [67,68,69,70].

The simplified figure used by Wüster, Modahl, and Arbuckle visually groups major lineages, but the underlying phylogeny and dataset do not use composite ‘early-diverging snake’ or Anguoidea terminals [14]. They analyse 4162 species-level tips and treat substantial uncertainty as unknown. Species-level resolution and explicit missing data are therefore strengths of the analysis, not defects. The remaining questions concern the evidentiary provenance of zero states, the biological information compressed into one observed character and one hidden precursor, and whether alternative characterisations of the non-caenophidian snake tips have been tested.

Tip misclassification has predictable topological effects in both directions. If weakly investigated lineages between or around positive taxa are entered as true absences, positive states become separated on the tree, and likelihood may be redistributed towards repeated gains, repeated short-lived precursors, or both. If incomplete or indirect positive observations are coded as certain functional venom, the inferred origin can be pushed deeper, and retention can be favoured over independent gains. Wüster, Modahl, and Arbuckle appropriately use unknown states for many unresolved taxa and code several focal anguimorph bridge species positively; their alternative schemes demonstrate fine-scale sensitivity within Anguimorpha [14]. They do not, however, test an alternative characterisation of the non-caenophidian snake tips central to the earlier-snake hypothesis. A future analysis should therefore retain their species-level resolution while adding a source-and-evidence ledger for both zero and positive states, probabilistic or unknown states where warranted, and an observation model that represents variation in research effort, detection, and false-positive classification.

Loss and reduction must also be treated as ordinary evolutionary outcomes. Comparative analyses across tetrapods have found that venom losses may occur at high rates relative to gains, and gene-family studies show that toxin status can be reversed [71,72]. At a finer scale, lineages can lose major toxin genes while retaining the rest of the venom system, as has been shown, for example, to occur in *Crotalus* species (rattlesnakes) [73]. Across toxicoferan reptiles, reduction in glandular complexity and toxin expression has been associated with alternative prey-subjugation mechanisms and dietary shifts [64,74,75].

The precursor model used by Wüster, Modahl, and Arbuckle explicitly allows functional venom to be lost either to the precursor state or to the non-precursor state [14]. Its reconstruction of multiple functional gains therefore cannot be attributed to omission of loss. A broader concern remains that one global transition process and one undifferentiated precursor category may not represent lineage-specific reductions or the several biological steps preceding functional venom. Models with unrealistically difficult loss can favour repeated gains, whereas models with unrealistically easy loss can preserve an ancient origin by scattering reversals across the tree. The remedy is to compare biologically explicit transition structures, inspect parameter identifiability, and test whether conclusions persist across plausible coding, topology, sampling, and detection assumptions [76].

A constructive reconstruction strategy would therefore retain the information contained in the four-event framework. It would code molecular availability, oral recruitment and secretion, functional venom, and delivery-system elaboration separately; use uncertain or probabilistic tip states where evidence is incomplete; model false-negative and false-positive observation error as a function of study design and evidence quality; allow reversal and reduction; preserve species-level terminals; and quantify support for alternative event histories through stochastic mapping, posterior event-count distributions, root-state conditioning, or an expanded state-history model. Ordered multistate models may capture a common progression, but they should not force every lineage through an identical anatomical sequence. Model adequacy and posterior predictive or simulation-based checks are as important as relative fit.

Ancestral-state reconstruction remains indispensable because the relevant ancestors cannot be observed directly and soft-tissue venom systems are rarely recoverable from fossils. Its output, however, cannot be stronger than the biological coding and transition structure supplied to it. The appropriate conclusion from a well-supported model is therefore conditional: given these definitions, these tip data, this phylogeny, and these allowed transitions, one history is better supported than its alternatives. That is a powerful statement when the conditions are explicit. It becomes misleading only when the conditions are mistaken for observations. The published reconstruction supports separate functional gains under its coding and model, whereas the evidence reviewed here identifies targeted tests of earlier functional origins. Because the present review does not fit an alternative model, it makes no claim that an earlier origin is better supported.

For the purposes of this manuscript, ‘origin’ will consequently be qualified throughout. An ancient shared molecular substrate is not yet a shared venom origin. Shared oral recruitment and secretion are stronger but still do not establish a selected venom role. A shared origin of functional venom requires evidence that the ancestral secretion was wound-delivered and fitness-enhancing; independent gland capsules, ducts, compressor muscles, or fangs represent later elaborations. Where those functional links cannot be tested or inferred from evidence-audited coding, the correct outcome is data deficiency, not forced assignment to either a single-origin or a multiple-origin narrative.

## 2. Origin and Evolution of Anguimorpha Lizard Venom Systems

### 2.1. An Early Functional-Origin Hypothesis with Parallel Elaboration of Delivery Architecture

Although functional venom remains incompletely demonstrated in several phylogenetically critical bridge taxa, the available glandular, molecular, and pharmacological evidence makes an early-origin hypothesis followed by retention, reduction, loss, and parallel gland elaboration in the *Heloderma* and *Lanthanotus*–*Varanus* lineages viable. However, the critical bridge taxa do not yet unite evidence of expelled secretion, natural wound delivery, realistic delivered dose, and a secretion-specific fitness benefit. This evidence therefore neither constitutes an ancestral-state test nor establishes functional venom at the anguimorph root, and it does not show that an early origin is historically better supported. Wüster, Modahl, and Arbuckle instead recover a single anguimorph precursor followed by multiple functional gains under all four coding schemes [14]. The alternative proposed here predicts that intervening Anguoidea should retain concordant combinations of derived, secreted, physiologically active components and homologous secretory cell states; this is a future test rather than a result of the present narrative review.

### 2.2. The Broad Inference Is Robust, While the Exact Anguimorph History Is Coding-Dependent

The immediate stimulus for revisiting this question is Wüster, Modahl, and Arbuckle’s ancestral-state analysis of squamate venom [14]. Its precursor models fit better than the direct binary models under all four coding schemes. Across those schemes, the analyses recover one key precursor within Anguimorpha followed by separate origins of functional venom in whichever clades are treated as venomous. The exact location of the precursor and the number and placement of subsequent functional gains change predictably when varanoids and other anguimorphs are reclassified. These are valuable sensitivity analyses: they demonstrate coding dependence in the fine-scale anguimorph history, but they do not overturn the principal inference of multiple functional gains. That inference remains conditional on the tested definitions and model structure rather than classification independent.

The underlying analysis uses 4162 species-level tips rather than aggregated anguimid terminals [14]. Of the twelve sampled *Abronia*, ten are unknown, while *A. graminea* and *A. lythrochila* are venomous in the first three schemes. All three sampled *Celestus* are coded as unknown. *Gerrhonotus infernalis* is venomous in the first three schemes, while two congeners are unknown. *Celestus warreni*, whose oral-gland evidence is discussed below, is absent from the tree; *Celestus* is now commonly placed in Diploglossidae [77].

These coding choices are appropriately cautious, but they do not resolve whether evidence from unsampled taxa such as *Celestus warreni*, or improved evidence from currently unknown congeners, would alter the reconstruction. Anatomy, derived toxin sequences, secretion, and pharmacology in the relevant lineages raise an explicit alternative characterisation [51,54]. A single early functional origin followed by reduction or loss therefore remains a testable hypothesis, but it cannot be preferred merely because it appears to require fewer novel recruitment events. Because the present review does not construct and compare an evidence-audited tip-level dataset, it treats that history as viable rather than better supported.

Ecological evidence remains especially difficult to obtain for weak, conditional, or prey-specific secretions. Failure to witness prey slowing or predator release cannot by itself distinguish true absence from a small effect, an unobserved delivery context, an inappropriate recipient, or limited observation. Wüster, Modahl, and Arbuckle did not simply convert the named anguimorph uncertainties into zeros; they used unknown and positive states where warranted [14]. The residual problem is that one binary observed state cannot record the quality of negative investigation or distinguish among anatomical, secretory, pharmacological, and ecological uncertainty. Those evidentiary layers should be represented explicitly in a future reconstruction.

This more permissive coding cannot be applied indiscriminately. A recent proteomic analysis of the mandibular glands of *Shinisaurus crocodilurus* detected no proteins that the authors could assign to a venom repertoire, and the species lacks grooved teeth [78]. This is consistent with prior work showing that this species has smaller seromucous glands with low levels of protein-secreting cells relative to other anguimorph lizards [51]. That is substantive present-day negative evidence, not merely a missing sample, and is data consistent with secondary loss. Under a one-origin model it would require strong reduction or loss; under a multiple-origin model it would count as an intervening absence. What it cannot establish alone is whether the negative state is primitive or secondary. The appropriate coding should therefore record both the quality of the negative investigation and the historical uncertainty.

Loss and reduction are well-documented features of venom-system evolution, rather than assumptions introduced solely to preserve a single-origin hypothesis. Toxin genes, expression profiles, and gland investment are labile when diet, prey-handling mechanics, defensive ecology, or energetic returns change. Comparative analyses across squamates document repeated gene-family expansion, contraction, and degeneration, and macroevolutionary models that permit both gains and losses generally fit venom histories better than irreversible-gain narratives [20,64,71,72]. Wüster, Modahl, and Arbuckle’s precursor model already permits functional venom to return to either the precursor or non-precursor state. The remaining quantitative question concerns support for the number and placement of gains and losses under biologically explicit state definitions and transition structures. A continuous-time Markov model represents transitions among discrete character states along phylogenetic branches using instantaneous rates; the Mk model is a standard family of such models for discrete characters. The Akaike information criterion (AIC) is a likelihood-based measure that compares model fit while penalising additional parameters. Earlier functionalisation followed by reductions and multiple later independent gains are alternative evolutionary histories, not automatically distinct models. Under a common fitted model and tip-state dataset, stochastic character mapping can estimate the posterior distributions of root states, gains, losses, and event timing. If the biological alternatives require different state spaces or transition constraints, those models must be specified explicitly and compared using appropriate likelihood-based or Bayesian criteria.

For Anguimorpha, the constructive modelling choice is consequently to separate at least four uncertainties: whether translated toxins occur in the oral gland, whether they enter material delivered through the bite, whether the delivered dose perturbs a relevant recipient, and whether that perturbation causally improves a fitness-relevant interaction. Taxa with positive evidence at the first three levels but no secretion-specific ecological contrast should not be collapsed with taxa in which a targeted anatomical and proteomic search is negative. Natural-history observations can inform priors, recipient choice, and assay design, but they do not substitute for a causal comparison, and their absence is not negative evidence. Models can then quantify support for alternative event histories (either earlier functionalisation followed by reductions, or multiple later independent gains) through stochastic mappings and event-count distributions, while allowing false-negative and false-positive classification and preventing uneven research effort or observational detectability from masquerading as evolutionary history.

### 2.3. The Anguoidea Bridge

The strongest evidence bearing on possible venom continuity across Anguimorpha is not the discovery of a single familiar transcript. It is the conjunction of gland anatomy, derived toxin repertoires, lineage-specific sequence innovation, evidence of translation and release into gland lumina, physiological activity, and a phylogenetic distribution linking the specialised endpoints (Table 1). The broad comparative study that established this framework sampled the full anatomical range from relatively simple mixed glands in anguids to encapsulated systems that evolved independently in the *Heloderma* and *Lanthanotus*–*Varanus* lineages [51]. In the simpler condition documented in Anguoidea, including the genera *Abronia*, *Celestus*, and *Gerrhonotus*, repeated mandibular compartments lie along the lower tooth row; serous protein-secreting tissue occupies the basal part of each compartment, mucus-secreting tissue lies above it, and lumina and drainage channels carry products towards openings adjacent to the teeth. In the specialised condition, mucus and protein glands are segregated, the protein gland is more heavily encapsulated, and storage spaces are enlarged. This anatomical series establishes continuity of a secretory substrate, which is suggestive of continuity of functional venom [51].

The study of the mandibular gland anatomy of *Abronia graminea* was particularly informative because the gland system is neither a rudimentary diffuse mucous field nor a miniature version of a helodermatid gland [54]. Its mandibular gland consists of robust, actively secretory, mixed seromucous compartments. Histological sections show secretory granules, enlarged vesicles, apparent exocytosis, luminal storage, and ducts oriented towards the tooth bases. The protein-secreting regions are larger and more intricately developed than the mucous-secreting regions. The deep-transcriptome survey recovered CRiSP, cystatin, kallikrein, lectin, B-type natriuretic peptide with helokinestatin domains, nerve growth factor, group III phospholipase A_2_, and Kunitz-family peptides, with molecular-evolution analyses identifying diversification in several toxin types, consistent with diversification within an oral secretory system, but does not test for fitness-relevant venom function [51].

The *A. graminea* study properly acknowledged a limitation: transcript recovery and secretory histology did not by themselves prove that every candidate was translated and present in secretion [54]. Proteomic work has since detected B-type natriuretic peptide, CRiSP, group III phospholipase A_2_, and kallikrein in mandibular-gland extracts from both *A. graminea* and *A. lythrochila*, with the same principal toxin classes represented at different abundances [79]. This closes an important transcript-to-protein gap and makes accidental low-level transcription less plausible. It does not yet measure the composition or dose expelled during a natural bite, and *A. lythrochila* has no grooved teeth; those limits bear on delivery efficiency and ecology, not on whether the gland translates a conserved suite of bioactive protein families.

The signal extends beyond *Abronia*. Mandibular-gland libraries from *Celestus warreni* and *Gerrhonotus infernalis* yielded overlapping CRiSP, kallikrein, natriuretic/helokinestatin, group III phospholipase A2, and other lineages. More importantly, some sequences carry innovations that are not predicted by a generic salivary-housekeeping explanation. The natriuretic precursor bears tandem, post-translationally liberated helokinestatin domains positionally homologous to those found in *Heloderma*; *Celestus* produces the novel proline-rich peptide celestoxin, which induced marked hypotension in experimental preparations; and homologous hyaluronidase sequences were recovered from *Gerrhonotus* and *Heloderma* [51]. Pancreatic-type ribonuclease and kallikrein lineages likewise display derived substitutions and cysteine-pattern changes. No single feature is decisive. Their co-occurrence in anatomically connected oral glands is compatible with inherited recruitment followed by divergence, but it must also be compared with a shared nonvenomous substrate followed by lineage-specific co-option.

At the specialised endpoints, the evidence for secreted toxic systems is direct. *Heloderma* venoms have a comparatively conserved overall proteomic core across species, even while component abundance varies, and independent proteomic surveys recover abundant kallikrein-family enzymes, exendins, helokinestatins, phospholipase A_2_, CRiSPs, and other proteins [80]. Function is broadly conserved: helodermatid venoms show kinin-generating and hypotensive activity, activate coagulation Factors XII and VII, bind voltage-gated sodium and calcium channel domains, depress neuromuscular transmission in ex vivo preparations, and produce intense pain and systemic effects in bites [81,82]. However, while the overall proteomic and functional traits are conserved, there is variation at the toxin level and functional diversification of individual toxins such as the exendins, helokinistatins, and natriuretic peptides, as well as functional variations in coagulotoxicity and neurotoxicity [51,81,82,83].

Varanoid evidence is broader but more heterogeneous. Comparative proteomics across twenty *Heloderma*, *Lanthanotus*, and *Varanus* species found ubiquitous but structurally and functionally variable kallikrein-family enzymes, together with interspecific changes in CRiSPs, lysosomal acid lipase, phospholipase A_2_, natriuretic peptides, and other components [84]. Several varanid secretions cleave fibrinogen destructively and impair clot formation; representative *Varanus* venoms also produce cardiovascular effects and bind ion-channel neuropathic targets [50,81,84,85]. These data establish a translated and active secretion but do not require every species to use it for the same ecological purpose.

The study of *Varanus komodoensis* integrated gland imaging, venom pharmacology, and finite-element analysis to argue that a relatively weak bite and pull-back feeding action work with anticoagulant and hypotensive secretion during predation [50]. That synthesis provides a mechanistic ecological hypothesis, although the magnitude and necessity of the venom contribution in free-ranging attacks remain to be fully elucidated. It is therefore evidence for plausible fitness benefit, not a licence to infer an identical role for all monitors.

*Lanthanotus borneensis* illustrates why state coding should remain explicit and revisable. Its six-compartment mandibular gland drains through separate ducts beside ungrooved teeth, and the gland secretion is rich in a biologically active kallikrein [84]. One study nevertheless concluded that calling the animal venomous was not then justified because neither a predatory nor defensive role had been demonstrated; it explicitly identified natural-prey experiments as the missing test [86]. However, subsequent work showed that *Lanthanotus* gland material cleaves kininogen and releases vasoactive kinins, extending a kallikrein-mediated pathophysiological mechanism across *Heloderma*, *Lanthanotus*, and multiple *Varanus* species [82]. That result strengthens the biological plausibility of functional continuity. The remaining uncertainty concerns the magnitude and fitness consequence of the secretion’s contribution under natural conditions, not whether an uncontrolled observation happens to record visibly impaired prey. A natural-prey test would be decisive only if a validated manipulation isolates secretion from mechanical injury, restraint, and stress; natural-history observation alone cannot supply that contrast.

### 2.4. Why the Repertoire Is Not Simply Salivary

The objection that oral-gland transcripts may simply be ordinary salivary or housekeeping genes is essential, because many protein families called ‘toxins’ have indispensable physiological roles outside venom glands. Comparative transcriptomic studies showed that homologues of numerous proposed venom genes occur in diverse python and lizard tissues, and that some early Toxicofera-wide recruitment claims conflated broadly expressed homologues with specialised toxin paralogues [16,17,18]. Those critiques rule out family-name matching as sufficient evidence and set the correct molecular standard: orthology and paralogy must be resolved, tissue enrichment established, translated products confirmed, and derived sequence or regulatory evolution linked to activity. However, it must also be noted that the well-characterised toxin types 3FTx and lectins were dominantly expressed in the python oral glands relative to other tissues [18].

The evidence for an early anguimorph functional origin becomes more informative when that stricter standard is applied, but it does not yet establish relative historical support. The underlying shared repertoire includes lineages recruited from physiological systems not restricted to saliva, including endocrine B-type natriuretic peptides, group III phospholipase A_2_, Kunitz-domain inhibitors, kallikreins, CRiSPs, and others [10,50,51,54,80,83,84]. Their mere presence in an oral library would be equivocal. The stronger evidence is that oral-gland sequences occupy derived toxin lineages, show tissue-associated expansion or episodic diversification, encode novel processing architectures, occur as proteins in gland material or expelled venom, and produce effects appropriate to a wounded recipient. This evidentiary conjunction was the basis of the original early-venom interpretation.

Derived precursor architecture is especially discriminating. In *Abronia*, *Celestus*, *Gerrhonotus*, and *Heloderma*, B-type natriuretic peptides occur in precursors whose propeptide regions de novo encode one or more proline-rich helokinestatins [51,54,83]. The natriuretic domain is comparatively conserved, whereas the helokinestatin region has undergone dramatic duplication, insertion, deletion, and diversifying sequence change and is post-translationally cleaved to liberate the peptides. Shared possession of this unusual multiproduct precursor is more informative than generic detection of a natriuretic transcript because it identifies a particular evolutionary modification of processing and product repertoire. It is compatible with inheritance from a common neoanguimorph ancestor followed by lineage-specific elaboration; repeated independent expression of an unmodified physiological gene does not predict it. In contrast, the propeptide region of the varanid natriuretic peptides lacks the helokinestatin peptides, revealing them to be a unique derivation that evolved after the varanoid split.

The informational value of these precursor-level characters is supported by the isolation and receptor pharmacology of helokinestatin and by biochemical evidence that helodermatid hyaluronidase acts as a spreading factor, observations that link molecular processing and secretion composition to recipient-directed effects [87,88].

Celestoxin makes the same point at a shallower scale. It is not simply a familiar toxin family relabelled after appearing in an oral transcriptome, but a novel precursor-derived peptide identified from a *Celestus warreni* oral-gland transcript and shown, as a synthesised peptide, to induce hypotension through vascular smooth-muscle effects [51]. Its restricted distribution indicates that the apparently modest gland is an arena of toxin innovation. This does not make celestoxin a synapomorphy of all anguimorph venom systems; rather, it shows that an intermediate lineage can inherit a secretory context in which novel bioactive products evolve, just as specialised *Heloderma* and *Varanus* lineages have generated their own derived components.

The data also contain an internal warning against overinterpretation. The *Abronia* transcriptome included a vascular endothelial growth factor sequence whose phylogenetic position was more consistent with an ordinary physiological homologue than a secreted venom recruitment [54]. Treating that transcript as a toxin merely because VEGF occurs in some venoms would inflate the repertoire. The same rule applies to CRiSP, kallikrein, phospholipase, Kunitz, ribonuclease, and natriuretic families: each candidate must earn toxin status through its particular phylogeny, sequence innovation, expression, translation, secretion, pharmacology, and ecological deployment. Some anguimorph components meet most of these criteria; others remain candidates.

Accordingly, ‘not simply salivary’ should not be read as ‘every detected protein is venom’. A conservative synthesis recognises a nested evidentiary pattern. *Heloderma* satisfies anatomy, expelled secretion, pharmacology, and bite-level effects. Multiple *Varanus* species satisfy anatomy, secretion proteomics, and target-specific pharmacology, with ecology varying by species. *Lanthanotus* satisfies gland anatomy and biochemical function but lacks a secretion-specific demonstration of fitness benefit. *Abronia* satisfies secretory anatomy, derived transcripts, and gland-extract proteomics, while delivered dose and secretion-specific natural-bite benefit remain unknown. *Celestus* and *Gerrhonotus* add derived sequences and, for celestoxin, direct pharmacology, but need modern proteomics and controlled ecological-performance tests. This gradation is broadly reflected in the coding used by Wüster, Modahl, and Arbuckle: selected *Abronia* and *Gerrhonotus* species are positive in the first three schemes, while most unresolved congeners and the sampled *Celestus* are unknown [14]. The outstanding task is to translate the evidentiary layers into explicit multistate or probabilistic characters, not to replace a purported blanket nonvenomous code.

### 2.5. Independent Lizard Gland Elaboration Does Not Require Independent Venom Origins

The lizard anatomical evidence supports two clear elaborations superimposed on a simpler shared pattern. The likely ancestral anguimorph mandibular condition comprised a row of relatively small mixed seromucous compartments: protein-secreting tissue basally, mucus-secreting tissue above, modest central lumina, thin connective-tissue capsules, and drainage towards the tooth bases. While most Anguoidea lineages have secondarily lost the maxillary glands, some still retain them [51]. Within the *Heloderma* lineage, mandibular gland compartments became enlarged and fused (reducing the number to six per side down from at least fifteen) and consolidated into a thickly encapsulated complex with extensive storage and delivery adaptations [51]. Separately, the ancestor of *Lanthanotus* and *Varanus* convergently evolved enlarged, thickly encapsulated protein glands, fused compartments (convergently down to six like *Heloderma*), expanded lumina, and segregation from mucus-secreting tissue [51]. The resemblance of the two endpoints is therefore convergent at the level of gland packaging and delivery efficiency. It is remarkable that both lineages reduced the total number of compartments to six, with the remaining compartments increasing posteriorly in size for both lineages [51].

Crucially, the encapsulated condition is unlikely to mark, or to have evolved concurrently with, the origin of venom. In the absence of a toxic secretion that improves fitness, there is no specific selection pressure for costly enlargement, encapsulation, storage, ducting, and segregation of protein-secreting tissue as a venom-delivery apparatus. The expected starting condition is therefore a simpler seromucous gland whose secretion first acquires a wound-delivered toxic benefit; only then can selection progressively improve its storage and delivery. The glands of Anguoidea such as *Abronia*, *Celestus*, and *Gerrhonotus* approximate this predicted intermediate condition, whereas *Heloderma* and the *Lanthanotus*–*Varanus* lineages represent later, parallel elaborations [51]. This architecture supports a functional-intermediate hypothesis, but it does not date the acquisition of a fitness-enhancing toxic secretion. The principal coding schemes of Wüster, Modahl, and Arbuckle also do not recognise only elaborate endpoints: selected *Abronia* and *Gerrhonotus*, as well as varanoids in applicable schemes, are coded as venomous [14]. Their separate functional gains therefore cannot be attributed to treating specialised delivery architecture as the sole venomous condition.

Architecture alone nevertheless cannot prove toxic function. Oral glands may be shaped by lubrication, digestion, antimicrobial defence, prey adhesion, oral-tissue maintenance, or several roles at once. Nor does anatomical complexity provide a universal clock: a lineage can retain an effective low-pressure secretion without a fang, and a large gland can be multifunctional. The architectural argument acquires evolutionary force only in conjunction with a translated and released repertoire of derived proteins, target-specific physiological activity at plausible concentrations, and evidence that the secretion reaches a bite wound. The strongest test of selected venom function is then a causal change in ecological performance demonstrated by a secretion-specific contrast, not a simple field observation that prey or a predator changed behaviour after a bite.

### 2.6. Heloderma: Defence, Predation, or Multifunctionality?

#### 2.6.1. Present Functions Are Plural: The Original Selective Driver Remains Unresolved

Wüster, Modahl, and Arbuckle describe *Heloderma* venoms as primarily defensive, rather than exclusively defensive [14]. Repeated field evidence that *H. suspectum* and *H. horridum* predominantly consume eggs, nestlings, and other vulnerable nest contents, together with severe pain and prolonged bite-and-chew delivery, makes defence a supported function. This evidence does not identify the interaction in which venom first evolved or establish identical selection across every species, age class, and ecological setting. Neuromuscular depression, binding to skeletal-muscle NaV1.4 and cardiac CaV1.2 channel domains, cardiovascular disruption, and activation of coagulation Factors XII and VII provide biologically plausible mechanisms by which venom could assist prey handling [81,82,83,85,89]. Direct demonstrations that naturally delivered venom improves capture success are nevertheless lacking. A predatory role is therefore mechanistically plausible but ecologically unverified, while the historical priority of either role remains unresolved.

#### 2.6.2. One Secretion, Several Evolutionary Questions

The customary question of whether *Heloderma* venom is for defence or predation compresses at least three separate problems. The first is current capacity: can the secretion deter an aggressor, compromise prey, or affect a conspecific? The second is current fitness benefit: does delivery actually improve survival, prey capture, handling safety, or competitive outcome under natural conditions? The third is historical causation: which interaction supplied the selection that originated the toxic secretion, and which later modified its composition and delivery? Evidence answering one problem cannot automatically answer the others. A venom system can undergo a major change in ecological emphasis, such as the ancestrally predatory venom of aculeate wasps subsequently being redeployed for defence in derived bee lineages [90,91,92]. In the case of the cone snails, they first evolved a predatory worm-specific venom, then a fish-specific defensive parallel venom, which was then co-opted in some lineages to be used as a fish-specific predatory venom with the secondary loss of the worm-specific toxins [93]. This underscores that the relative importance of those functions can change among descendants.

This distinction is especially important in *Heloderma*. Its mandibular venom glands are large, encapsulated, and divided into storage-rich compartments [51], but delivery is not the rapid, high-pressure injection associated with many front-fanged snakes. Multiple ducts open beside the lower teeth, and venom enters a wound as the lizard maintains its grip and chews, allowing secretion to track along grooved teeth. The apparatus can therefore serve in a prolonged defensive bite, but the same mechanics can expose a restrained prey item to a physiologically meaningful dose. Anatomy identifies an efficient wound-delivery system; it does not identify the recipient against which that system was first selected.

Nor should the venom be treated as a unitary adaptation merely because it is delivered as one secretion. Proteomic and transcriptomic studies of *Heloderma* venoms recover a chemically diverse mixture that includes kallikrein-family enzymes, exendins, helokinestatins, natriuretic peptides, CRiSPs, helofensins, phospholipase A_2_, hyaluronidase, and additional enzymes and inhibitors [51,80,83,94]. Distinct components can act on pain pathways, vascular tone, cardiac conduction, haemostasis, or neuromuscular transmission, and they need not have been recruited at the same time or maintained by the same ecological interaction. The unusual domain duplications and processing innovations documented in helofensins, helokinestatins, and exendins make that mosaic history explicit [83].

Individual *Heloderma* products also have well-resolved biochemical histories. The Exendin-3 and exendin-4 peptides (which contain the glucagon-peptide domains) are less potent hypotensive agents than the derived exendin-1 and exendin-2 (which contain the vasoactive-intestinal-peptide domain), further illustrating the processing diversity hidden within the whole secretion [83,95,96].

#### 2.6.3. Aposematism Is a Species- and Context-Specific Hypothesis

The striking black-and-orange, pink, or yellow pattern of many *Heloderma suspectum* individuals makes a warning-signal interpretation plausible. The error is to convert that plausible species-level interpretation into a genus-wide premise. Modern treatments recognise *H. suspectum* alongside four deeply differentiated beaded-lizard species (*H. alvarezi*, *H. charlesbogerti*, *H. exasperatum*, and *H. horridum*) whose colour characters and ontogenetic trajectories are not interchangeable; most species other than *H. suspectum* appear to be cryptically coloured [97]. In particular, *H. alvarezi* undergoes pronounced ontogenetic melanism: juveniles can retain yellow spotting and banding that is largely or entirely lost in dark, cryptically coloured adults. The beaded-lizard lineages more generally span patterns that can be substantially darker and lower in contrast than the familiar Gila-monster phenotype.

Conspicuousness also is not an intrinsic property of a colour swatch. A signal is conspicuous to a particular viewer against a particular background, at a particular distance, under particular illumination, and during particular behaviour. Demonstrating aposematism therefore requires more than a simplistic human judgement that an animal looks bright: it requires evidence that relevant predators detect the pattern, associate it with an aversive consequence, and alter their behaviour in a way that benefits the signaller. Signal efficacy can also be distance-dependent, with the same pattern contributing to crypsis at one scale and warning at another. These principles are well established in the warning-colour literature [98].

The prediction that warning-signal efficacy will depend on the predator community and visual environment is also supported by comparative theory and experiments showing that different predator assemblages can alter the conspicuousness and advantage of aposematic signals [99].

The appropriate conclusion is not that *H. suspectum* is non-aposematic. Its pattern, defensive postures, and painful bite together make aposematism a strong testable hypothesis. The narrower conclusion is that the hypothesis cannot be generalised from *H. suspectum* to all *Heloderma* without predator-vision modelling and behavioural tests across species, life stages, substrates, and predator guilds. Even a confirmed warning signal in *H. suspectum* would establish a present association between appearance and defence, not the age of that association. Warning colouration can evolve after a noxious defence already exists, and a venom first maintained in another context can subsequently become the punishment advertised by a visual signal.

#### 2.6.4. Pain Establishes Defensive Capacity, Not Evolutionary Primacy

The defensive evidence is strongest at the level of consequence. Human envenomations are uncommon, but the clinical literature repeatedly records immediate or severe pain, local swelling, paraesthesia, nausea, vomiting, diaphoresis, weakness, hypotension, tachycardia, and, in the most serious cases, angioedema, conduction abnormalities, shock, coagulopathy, or myocardial injury [100,101,102,103,104,105,106,107]. Most bites are mild to moderate, but a small subset requires emergency resuscitation, and a confirmed fatality is on record [105]. A large poison-centre analysis provides additional clinical support while also documenting heterogeneity in outcome and management across reported *H. suspectum* envenomations [108].

Pain is not restricted to conspicuously patterned *H. suspectum*. For example, a well-documented bite by a wild juvenile Guatemalan beaded lizard, then identified as *H. horridum charlesbogerti* and now treated as *H. charlesbogerti*, produced severe local pain, paraesthesia, vomiting, diaphoresis, dizziness, and marked hypotension after an attachment of approximately fifteen seconds [109]. The observation is important because it separates the capacity to cause painful defensive envenomation from the bright-coloured Gila-monster phenotype to the cryptically patterned *H. charlesbogerti* phenotype.

These observations establish efficacy against a large mammal and make predator deterrence biologically credible. However, they do not reveal why venom originated. Humans are not natural prey or predators, clinical bite reports do not sample a defined or historically consistent predator class, many reported bites occur during capture or handling, and symptom severity is strongly affected by bite duration, attachment, site, victim physiology, and delivered dose. The mechanical trauma of a sustained bite also contributes to pain. A human case series can therefore demonstrate what the whole apparatus is capable of doing, but it cannot estimate the selection coefficient imposed by natural predators or compare that coefficient with benefits obtained during feeding.

The biochemical basis of pain is likewise plural. Kallikrein-family enzymes in *Heloderma* venom cleave kininogen and release vasoactive kinins [82,110,111,112], a mechanism expected to promote pain, vasodilation, increased vascular permeability, hypotension, and oedema. Other components may modify sensory or ion-channel activity, and cardiovascular stress can amplify the aversive experience. A painful clinical syndrome is therefore not a single-purpose toxin phenotype. Kinin release can deter a predator, but hypotension and systemic disruption can also reduce the capacity of an active prey item to resist or escape. The hypotensive effects of exendins and natriuretic peptides [51] would potentiate this effect. The relevant evidence supports defence as one function; it does not make all contributing molecules defensive adaptations.

#### 2.6.5. Neurotoxicity Provides a Credible Predatory Mechanism

The strongest challenge to a defence-only interpretation is the evidence for paralysis. Earlier work showed that purified helothermine from *H. horridum* can interfere with ryanodine-receptor-associated calcium handling and produce lethargy and hind-limb paralysis in experimental rodents [113,114,115,116]. In addition, the protein class originally called lethal toxin-1 and later recognised as the multidomain β-defensin-derived helofensins inhibited electrically stimulated mammalian skeletal muscle [83,89]. Those findings left the natural target and ecological role incompletely resolved, but they established that the venom contains more than algesic or hypotensive activity. The molecular diversification of helofensins also identifies them as derived products rather than incidental salivary homologues [83].

A direct comparative investigation subsequently tested crude venoms from *H. exasperatum*, *H. horridum*, and *H. suspectum* in chick biventer cervicis nerve–muscle preparations [81]. All three reduced nerve-mediated twitch responses after an initial contraction, with preserved responses to postsynaptic agonists and no evidence of direct myotoxicity in the preparation, a pattern consistent with presynaptic neurotoxicity. In parallel biolayer-interferometry assays, *H. suspectum* venom bound mimotopes representing the S3–S4 extracellular loop of voltage-sensing domain IV of human skeletal-muscle NaV1.4 and cardiac L-type CaV1.2 channels [81]. The interspecific pattern is suggestive. *H. horridum* and *H. exasperatum* were comparatively potent in the avian neuromuscular preparation, whereas *H. suspectum* showed stronger binding in the mammalian channel-domain assays. Because all three species can encounter avian and mammalian food, such divergence is compatible with prey-specific selection on toxin isoforms or abundances. A toxin that depresses skeletal-muscle activation can reduce struggling or escape, and a toxin that perturbs cardiac calcium-channel function can impose an additional physiological cost. Neither action must kill rapidly to be beneficial: a modest decrease in prey performance during a close-contact feeding event may reduce handling time or injury risk.

The limits of this evidence should be stated as clearly as its force. A chick nerve–muscle preparation is not a natural predation trial, and binding to an isolated channel-loop mimotope is not equivalent to demonstrating modulation of an intact channel in a living prey animal. Crude-venom concentrations in vitro do not by themselves establish the dose delivered in a bite, the distribution of that dose, or the time course relative to prey escape. Fractionation is still required to identify the responsible molecules, and intact-channel electrophysiology is needed to connect binding with gating effects. These results justify testing a predatory contribution to: ascertain if paralysis improves capture under natural conditions; identify the selection that produced the activity; or exclude a contribution of systemic disruption to defence.

#### 2.6.6. Cardiovascular Disruption Crosses Ecological Categories

Cardiovascular activity further resists a one-label defensive-use interpretation. Tachycardia, arrhythmia, hypotension, and occasional ischaemic presentations occur in severe human envenomations [104,106,107]. The NaV1.4 and CaV1.2 binding results offer one mechanistic route, while kinin generation, natriuretic peptides, helokinestatins, exendins, and other vasoactive components provide additional and potentially synergistic routes [51,81,83,87,117]. Experimental administration of *H. horridum* venom to rats has also produced electrocardiographic alterations, including changes in pacemaker activity, ectopic beats, and conduction, reinforcing that cardiac effects are genuine rather than merely inferred from human distress [118].

These effects could strengthen defence by making an encounter memorable and physiologically costly. They could equally assist predation by weakening a mammal or bird held in the jaws. The alternatives are not mutually exclusive, and the recipient need not experience every component in the same way. A rapid local pain signal may dominate predator deterrence, whereas systemic hypotension or cardiac impairment may become more consequential during a sustained prey bite. Present-day whole-venom activity can therefore be shared even if the histories of its component traits differ.

#### 2.6.7. Factor XII and Factor VII Activation Are Genuine Procoagulant Toxicity

The coagulotoxicity evidence adds a separate physiological axis with potential relevance to prey handling. Thromboelastographic assays showed that all tested *Heloderma* venoms were procoagulant, accelerating clot formation in mammalian and avian plasma [82,119]. Purified-zymogen assays then localised activity to activation of Factor XII and Factor VII, while finding no corresponding activation of Factor XI. This distinction matters. The result is not merely an association between venom and an altered clot trace, nor the pseudo-procoagulant production of weak fibrin by nonspecific proteolysis; it demonstrates direct activation of upstream clotting factors capable of initiating the intrinsic/contact and extrinsic pathways. Whether the resulting pathology in a naturally bitten animal is focal thrombosis, systemic dysregulation, factor consumption, or a more modest loss of performance will depend on delivered dose and host physiology. The secretion therefore possesses genuine procoagulant toxicity that is compatible with predatory function.

There is also structured interspecific and ontogenetic variation in coagulotoxicity [82]. Adult *H. suspectum* and neonate *H. exasperatum* venoms were more potent Factor XII activators than adult *H. exasperatum* and adult *H. horridum* venoms, whereas adult *H. horridum* was the most potent Factor VII activator. Because birds lack Factor XII but retain the extrinsic pathway involving Factor VII, the distribution of activities is compatible with differing exposure to mammalian and avian prey. The resemblance between adult *H. suspectum* and neonatal *H. exasperatum* also raises an explicitly ontogenetic hypothesis: larger beaded lizards may shift toxin isoforms or regulatory emphasis as body size, habitat use, and diet change.

Factor XII and Factor VII activation, and their interspecific and ontogenetic variation, are compatible with prey-related selection and justify direct ecological testing. Differentiated activity against clotting pathways may reflect prey-specific adaptation.

#### 2.6.8. Diet Records Do Not Settle the Question

The traditional argument against a predatory role begins with diet. *Heloderma* species are expert nest raiders and consume eggs, nestlings, and neonatal vertebrates, prey that may be unable to flee or defend themselves. Long-term field studies of *H. suspectum* in Utah and *H. horridum* in Jalisco established the seasonal importance of vertebrate nests, and functional work on *H. horridum* documented ovophagous feeding mechanics for breaching and processing eggs [120,121,122].

Predatory behaviour provides intriguing but non-decisive context. Experimental *H. suspectum* studies show prolonged post-bite elevation in tongue-flicking and strike-induced chemosensory searching, behaviours that can help relocate bitten prey [123]. Such searching is not unique to venomous reptiles and does not prove that *H. suspectum* releases prey to await envenomation. It does, however, demonstrate a behavioural system capable of exploiting chemical information after a bite and cautions against portraying the animal as exclusively an egg consumer with no relevant interaction with mobile prey.

The decisive ecological experiments have not yet been performed. Under appropriate welfare and ethical controls, they would compare intact venom delivery with validated sham, secretion-blocked, depleted, or pharmacologically neutralised conditions during encounters with ecologically relevant mammalian and avian prey. The manipulation must be shown not to alter bite mechanics, motivation, restraint, or prey stress. The delivered dose should be measured, and pre-specified outcomes should include immobilisation time, escape distance, handling duration, injury to the lizard, and capture success. Comparable predator assays should quantify withdrawal, learned avoidance, and survival benefit rather than relying only on human pain reports. Ontogenetic studies should pair venom from known individuals with quantified diet and habitat use. Until such data exist, natural history can identify relevant interactions and guide experimental design, but it neither disproves predation nor identifies defence as the predominant role.

#### 2.6.9. Competition Broadens the Functional Landscape

A strict defence-versus-predation dichotomy also omits interactions among conspecifics. Captive trials involving adult female *H. suspectum* documented intense female–female aggression that included sustained biting, envenomation, and lateral rotation; bitten animals were behaviourally and physically compromised before recovering [124]. The trials were few, conducted in captivity, and do not establish how often venom influences territoriality, nest access, or reproductive competition in nature. They nevertheless demonstrate a third recipient class in which delivery can have an immediate functional consequence.

Competition could influence venom maintenance if comparable interactions occur naturally, but the present captive observations establish capacity rather than the frequency or fitness consequences of conspecific envenomation in the wild. They broaden the recipient classes and outcomes that future studies should test without establishing competition as a current ecological function.

#### 2.6.10. A Testable Multifunctionality Hypothesis

The chemical and pharmacological diversity of *Heloderma* venom supports a testable multifunctionality hypothesis rather than an established defensive ecological conclusion. Kallikrein-mediated kinin release and other algesic effects can make a defensive encounter rapidly aversive. Hypotension produced by kinin release and vasoactive peptides could impair a bitten animal; neuromuscular depression and NaV1.4-associated activity could reduce motor performance; CaV1.2 binding and vasoactive components could disrupt cardiovascular function; and Factor XII and Factor VII activation could perturb haemostasis. These capacities may overlap among predators, prey, and conspecifics, but assays of physiological activity do not identify the natural recipient, delivered dose, or fitness benefit. A comparative study appropriately described the broader evolution of anguimorph venoms as dynamic and multifunctional [84]; for *Heloderma*, each proposed additional function still requires direct ecological confirmation.

#### 2.6.11. Different Effects Can Have Different Histories

Multifunctionality does not mean that every toxin performs every role or that all roles are equally important. It is a historical hypothesis about a composite system. An ancestrally predatory component can be co-opted for defence when its target is conserved in a predator; an ancestrally defensive algesic component can contribute to prey handling; and a component recruited for one physiological endpoint can later diversify under a different recipient. The conserved high-level venom profiles reported across *Heloderma* do not contradict this model, because changes in isoforms, relative abundance, processing, and target specificity can alter function without replacing entire protein classes. The cardiotoxic, coagulotoxic, and neurotoxic comparisons already indicate such functional differentiation beneath a broadly conserved proteomic scaffold. As such the venom may be multifunctional (Figure 2).

Accordingly, severe pain justifies the statement that defence is an important present function, but it does not justify the stronger claim that defence originated the venom or dominates selection across the genus. Paralysis, channel binding, cardiovascular disruption, and genuine procoagulant toxicity make a predatory contribution biologically credible, and interspecific and ontogenetic variation supply testable prey-related predictions, but these data stop short of demonstrating a natural capture advantage. Competition is a plausible additional role suggested by captive interactions. The present phenotype is therefore best described as multifunctional; the identity of the original or predominant selective driver is, at present, data deficient. The resulting evidential asymmetry (conspicuous and readily reported defensive effects, contextual diet evidence that does not measure venom delivery, and mechanistically plausible predatory effects in encounters that are difficult to observe) is summarised in Figure 2.

## 3. Snake Venom Origin and Evolution

### 3.1. An Earlier Distributed Functional Secretion as a Viable Hypothesis, with Later Maxillary Elaboration and Lineage-Specific Reduction

Evidence outside Caenophidia makes an earlier functional origin a biologically explicit and viable alternative to a Caenophidia-only origin. The positive evidence is distributed across different taxa. Taken together, the observations are compatible with a low-output venom system near the base of Alethinophidia, expressed through distributed maxillary, mandibular, and rictal secretory tissues. Under this scenario, colubroideans subsequently concentrated the maxillary component and segregated the inferred ancestral mixed seromucous condition into distinct protein-secretory and mucous glands, accompanied by encapsulation, storage, and directed ducting of the protein-rich component. In the absence of a secretion producing a fitness-relevant toxic effect, it is unlikely for there to be a specific selection pressure for the coordinated evolution of these costly features as a venom-delivery apparatus. As in anguimorphs, this derived architecture supports a functional-intermediate hypothesis. Conversely, powerful constrictors within the boid and pythonid radiations may have secondarily reduced toxin production as mechanical prey subjugation became dominant and mucus acquired greater importance in handling and swallowing large prey [48,64]. However, several other early lineages remain inadequately sampled, historical functional reports require replication, and direct proteomic and functional confirmation is still needed from anatomically identified *Cylindrophis* secretions.

### 3.2. The Inferred Caenophidia-Only Origin Is Conditional on Untested Non-Caenophidian Coding

The proposition by Wüster, Modahl, and Arbuckle that snake venom originated within Caenophidia is presented as an anatomical observation: early-diverging snakes lack a discrete encapsulated venom gland that is present in caenophidians [14]. That comparison changes the character midway through the argument. It identifies the origin of a conspicuous delivery apparatus, not necessarily the first origin of a toxic oral secretion delivered into a wound. Just as for lizards, a secretion in snakes released from comparatively simple serous or seromucous glands during biting and holding can satisfy the functional requirements of venom before a large encapsulated gland, a dedicated compressor muscle, a duct terminating beside a specialised fang, or forceful injection has evolved. Absence of those later features is therefore not equivalent to absence of the earlier function.

This distinction is consequential in the ancestral-state analysis that provided the immediate stimulus for the present review. Wüster, Modahl, and Arbuckle used 4162 species-level tips rather than a broad analytical terminal, but all sampled *Cylindrophis*, *Eryx*, boids, pythons, and other relevant non-caenophidian snakes were coded as nonvenomous across the four schemes [14]. The evidentiary certainty of those zero states varies, especially where anatomical, secretion, dose, and ecological tests are incomplete. The published sensitivity analyses therefore do not test the alternative characterisation advanced here. This does not invalidate their reconstruction: it accurately shows what follows from the supplied states and transition model. It does mean that the timing of snake functionalisation remains conditional on coding less specialised maxillary, mandibular, and rictal systems as nonvenomous.

The problem is not resolved solely by replacing a direct venom/no-venom model with one hidden precursor state. A precursor can mean a protein-secreting oral gland, oral enrichment of physiological homologues, a secreted but weakly active mixture, or a system close to a fitness-relevant toxic function. Those conditions are not interchangeable. In the supplied data, the relevant non-caenophidian observations enter as species-level zeros rather than as an aggregated terminal. An earlier origin followed by reduction can therefore be evaluated only after the anatomical, proteomic, pharmacological, and ecological evidence is translated into evidence-audited alternative tip states. This is a constructive limitation rather than a rejection of ancestral-state reconstruction: the output is conditional on the biological state definitions, evidence quality, and transition structure supplied to it. Both positive and negative tip assignments should carry probabilities that reflect their study designs.

### 3.3. The Phylogenetic Question Spans Multiple Non-Caenophidian Lineages

Non-caenophidian snakes do not form one biologically uniform residue. *Cylindrophis* and uropeltids belong to early alethinophidian radiations within Uropeltoidea; sand boas such as *Eryx* occur within Booidea; and pythons, including *Aspidites*, represent another macrostomatan lineage outside Caenophidia. Modern species-level and phylogenomic analyses differ in some deep relationships but consistently place these taxa on distinct branches [125,126,127]. Wüster, Modahl, and Arbuckle already represent that diversity with species-level tips [14]. Its importance is therefore biological and phylogenetic: concordant positive evidence across separated branches would carry more information about ancestral function than evidence from one lineage, but it must first be coded and analysed explicitly.

That distribution matters because each securely established positive lineage would change the minimum historical claim. Homology of the caenophidian venom gland places the underlying secretory system before the diversification of rear-fanged and front-fanged caenophidian apparatuses and therefore before Colubroidea as conventionally circumscribed. A genuinely functional secretion in a python would push the condition outside Caenophidia, while concordant evidence in *Cylindrophis*, uropeltids, and *Eryx* would strengthen the evidentiary basis for testing venom at least at the base of Alethinophidia, but historical placement would still require an explicit transition model. Secure positive evidence from Scolecophidia would provide an unusually informative test of extending the origin to the root of Serpentes, but the converse does not follow. Scolecophidia are early-diverging yet highly derived in skull form, gape, fossoriality, and specialised feeding on social-insect brood [49]; even a well-supported modern absence could be secondary due to an egg-eating lifestyle, as has been seen in egg-eating sea snakes [74,75] and egg-eating colubrids [48]. Reports in Scolecophidia of large serous glands beneath the eye further make a blanket nonvenomous code premature [49]. The root-of-Serpentes hypothesis is therefore data deficient, not excluded.

### 3.4. Cylindrophis Is the Critical Anatomical Taxon

Among non-caenophidian snakes, *Cylindrophis ruffus* is currently the most important anatomical and molecular bridge. Detailed dissection and histology reveal a circumlabial system more extensive than a single undifferentiated salivary field. The upper jaw contains a well-developed maxillary gland, the lower jaw a mandibular gland, and the corner of the mouth large superior and inferior rictal glands [64,128]. Histology, magnetic-resonance imaging, and molecular sampling demonstrated that the maxillary and mandibular components are mixed seromucous tissues with conspicuous protein-secreting cells and luminal drainage, while the rictal components are strongly serous and discharge through ducts near the rictus [64].

Anatomical distribution is not an objection to a venom interpretation. It is a plausible ancestral condition. The specialised caenophidian gland occupies the upper-jaw field, but comparative snake anatomy retains glandular tissues around much of the lip margin and rictus. A distributed ancestral system could release secretion from several openings into the oral cavity and bite wound during prolonged biting or holding; selection for more reliable delivery could later increase the size, encapsulation, storage capacity, ducting, and muscular compression of one component. On that model, the maxillary, mandibular, and rictal glands of *Cylindrophis* are not imperfect versions of a viper gland. They are candidates for an earlier organisation from which one region was subsequently privileged.

Nor does gland anatomy alone prove venom. Serous proteins can aid digestion or antimicrobial defence, mucus can lubricate prey, and any oral epithelium must maintain its own physiology. The evolutionary force of the *Cylindrophis* anatomy lies in conjunction: large and differentiated protein-secreting compartments occur in the same animal in which related, derived candidate toxins are expressed across the oral regions. Histology establishes a plausible production and release apparatus; it does not by itself establish recipient-directed toxicity or fitness benefit. That distinction is essential because the early-origin hypothesis should stand on layered evidence rather than on relabelling every serous gland as a venom gland.

The central molecular observation is not simply that familiar toxin-family names can be attached to oral transcripts. Identical candidate-toxin transcripts were recovered from maxillary, mandibular, and rictal samples of *Cylindrophis ruffus* [64]. These included characterised toxin types such as three-finger toxins, CRiSPs, a complement C3/cobra-venom-factor-like sequence, lectins, and veficolins. Repetition of the same isoforms in anatomically distinct oral compartments is more consistent with coordinated oral deployment or shared regulatory history than with four wholly unrelated local secretions. It supports treating the tissues as an integrated oral secretory system, even though it does not determine the ecological target of every component.

The three-finger-toxin sequences contain a more discriminating signal. *Cylindrophis* and the pythonid *Aspidites melanocephalus* possess derived three-finger-toxin isoforms with an additional cysteine immediately preceding the ancestral terminal cysteine pair, producing an unusual eleven-cysteine scaffold which may enable toxin dimerisation [64]. One *Cylindrophis* 3FTx molecular lineage also exhibits an exon deletion that yields a novel six-cysteine peptide. Such innovations are more informative than the generic detection of a three-finger-toxin superfamily transcript because they identify lineage-specific modification of a secreted-protein scaffold. They are compatible with active diversification in an oral system rather than mere unaltered expression of a physiological housekeeping homologue. The same study recovered evidence of differential duplication, diversification, and degeneration across other toxicoferan oral systems, underscoring that gain and loss are expected parts of the history rather than exceptions [64].

Nevertheless, repertoire annotation must be conservative. CRiSPs, lectins, complement proteins, and three-finger-protein relatives all have nonvenom physiological histories, and similarity to a toxin does not convert an oral transcript into a toxin. The strongest identification would combine phylogenetic placement of a derived paralogue, conserved synteny, novel precursor or mature-peptide architecture, oral-cell enrichment relative to other tissues, translation, release into secretion, activity at realistic concentrations, and benefit during natural delivery. The *Cylindrophis* dataset satisfies important anatomical, expression, and sequence-innovation layers, but not all of them. Its value is therefore substantial without being definitive.

### 3.5. Secretion Outside Caenophidia

In *Aspidites melanocephalus*, liquid chromatography tandem mass spectrometry recovered the dominant three-finger toxin and lectin products predicted from the oral-gland transcriptome. This is direct evidence that candidate toxins can be translated and released in the oral secretion of a non-caenophidian snake [64]. Immunological work also found cross-reactivity between *Aspidites* oral secretion and reagents used in a commercial venom-detection system, although such cross-reactivity is only corroboration of shared antigenic proteins and cannot establish toxic function [129].

This comparative result closes one evidentiary gap but leaves two others open. It establishes secretion outside Caenophidia in a pythonid; it does not demonstrate that the secreted *Aspidites* proteins perturb a recipient at naturally delivered concentrations, and it does not substitute for proteomics of *Cylindrophis* secretion. The current rationale for testing an early-alethinophidian origin therefore joins complementary observations made in different taxa: extensive serous anatomy and derived oral isoforms in *Cylindrophis*, translation and secretion in *Aspidites*, and historical functional effects in *Eryx* and uropeltids. Because these layers are not yet demonstrated in the same species, their combination is vulnerable to false-positive classification and cannot at this time conclusively establish functional venom near the root. A future study that unites all layers in the same non-caenophidian species would provide a direct test rather than a cross-lineage synthesis.

Functional observations outside Caenophidia predate modern toxinology. Phisalix and Caius reported that material attributed to the rictal or oral glands of the sand boa *Eryx conicus* caused paralysis and toxicity in birds, and they reported toxic effects from comparable secretions in six uropeltid species [130]. These experiments predate contemporary standards for gland identification, protein characterisation, dose normalisation, blinding, controls, and statistical analysis. The material administered, route of exposure, delivered dose, and reproducibility are not documented at the level now required to establish a venom function. Those limitations justify lower confidence and an uncertain state rather than automatic conversion of the reports into either confirmed venom or confirmed absence. An evidence-weighted coding should record them as directionally positive but with a degree of uncertainty. If the effects arose from contamination, nonphysiological dosing, or misidentified tissue, modern replication should fail; if they reflect a retained low-output venom, replication should recover gland-specific secreted proteins and dose-dependent activity. Because the relevant tips are coded as nonvenomous in all four of Wüster, Modahl, and Arbuckle’s models, a dedicated sensitivity analysis would be needed to determine whether an evidence-weighted treatment of these reports changes the reconstruction [14].

A decisive modern experiment is technically straightforward but must link the evidence layers. Maxillary, mandibular, superior-rictal, and inferior-rictal tissues should be identified histologically and sampled separately, with nonoral tissues and mucous-dominant oral regions as controls. Quantitative transcriptomics and spatial methods should establish cell-specific enrichment; bottom-up and top-down proteomics should confirm mature products, post-translational processing, and absolute abundance in freshly collected secretion. Purified or recombinant isoforms should then be tested at secretion-realistic concentrations in neuromuscular preparations and other physiological assays selected from the predicted molecular targets. Assays should include ecologically relevant vertebrate and invertebrate prey rather than relying on one non-target laboratory species.

The final test is delivery and fitness consequences. Naturalistic bite trials, conducted under appropriate welfare and ethical controls, should compare intact secretion with validated secretion-blocked, depleted, or neutralised conditions, matched sham treatment, and matched mechanical injury. The manipulation must leave bite force, tooth penetration, bite duration, jaw movements, handling, and prey condition otherwise unchanged. Endpoints should include immobilisation time, prey escape, handling duration, injury to the snake, and capture probability, with delivered dose quantified where possible. Simple field observation of prey impairment, or its absence, cannot isolate venom from the rest of the bite-and-capture sequence and should not be treated as categorical evidence. Equivalent predator-deterrence assays would be needed if defence is proposed, while conspecific assays would be needed for competitive or sexual functions.

### 3.6. Powerful Constrictors Fit a Secondary-Reduction Model

Boas and pythons are often treated as decisive negative taxa because they subdue prey by constriction and lack a caenophidian-style venom apparatus. An early-origin reconstruction makes a different, testable prediction. Once powerful constriction provides rapid mechanical incapacitation, the marginal benefit of chemical immobilisation can decline. Selection can then tolerate reduced toxin transcription, degeneration or loss of toxin paralogues, diminished serous storage, and a shift towards mucous secretion [64]. Increased mucus would remain useful for lubricating large or resistant prey during oral transport and swallowing even if recipient-directed toxicity were reduced. The predominantly mucoid oral glands and low-output candidate-toxin profiles reported in representative pythonids are compatible with that transition [64]. This is consistent with the atrophying of venom glands in the constricting colubrid lineages such as *Pantherophis* [48].

The polarising character is not simply ‘mucous gland present’. Comparative morphology indicates that predominantly serous or mixed seromucous labial tissues are closer to the plesiomorphic snake condition, whereas the enlarged, predominantly mucous glands of boas and pythons are derived [48,64]. Their anatomy is readily explained by selection for lubrication after constriction enabled the handling and ingestion of large prey and relaxed selection on chemical immobilisation. Consequently, failure to demonstrate functional venom in a boid or pythonid would be consistent with secondary reduction; it would not establish that the snake ancestor, or the common toxicoferan ancestor, lacked venom.

The strongest molecular counterargument comes from tissue-wide expression studies. In the Burmese python, many homologues of gene families commonly called venom toxins are expressed not only in the rictal gland but also in the brain, kidney, liver, gonad, small intestine, and other tissues; for several genes, expression outside the oral gland is comparable to or greater than oral expression. Reyes-Velasco and colleagues therefore argued that much of the supposed ancestral venom repertoire consists of broadly expressed physiological genes and proposed a model in which extensive duplication, restriction to the venom gland, and neofunctionalisation occurred chiefly in the advanced-snake lineage [18]. Comparative transcriptomic critiques have similarly shown that toxin-family labels can be generated by incomplete taxon sampling, short assemblies, or failure to distinguish one-to-one physiological orthologues from venom paralogues [16,17].

That critique should be accepted at its proper level: transcript presence alone is not evidence of venom. It does not follow that every orally expressed protein outside Caenophidia is physiological or that the entire circumlabial system is irrelevant to venom ancestry. Four distinctions remain available. A physiological homologue can be expressed weakly in many tissues while a derived paralogue is strongly enriched in a particular oral cell type; a protein can be transcribed without being secreted; a secreted protein can be inactive at realistic concentrations; and an active secretion can affect a laboratory preparation without improving a natural bite. Tissue-wide transcriptomics addresses the first distinction but cannot, by itself, decide the others.

The present early-origin case therefore does not rest on the claim that every python ‘venom-gene homologue’ is a toxin. It rests on the combination of extensive serous anatomy and coordinated oral expression in *Cylindrophis*, derived sequence innovation in non-caenophidian three-finger-toxin lineages, direct secretion of dominant transcript products in *Aspidites*, and historical toxicity reports from *Eryx* and uropeltids. None of these layers is individually decisive, and the layers have not yet been united in one species. Together, however, they make ‘all early snakes are simply nonvenomous’ a stronger claim than the data warrant. The appropriate contrast is not between a proven early venom and a proven late venom, but between an early-origin hypothesis with several independent positive signals and a late-origin reconstruction that depends partly on coding those signals as absent.

### 3.7. Secondary Reduction and Loss of the Venom System Within Caenophidia

Reduction is not an ad hoc device invented to rescue an early origin. Venom systems repeatedly lose complexity when ecology changes, such as a switch to egg-eating or constriction as a mode of prey capture. The clearest cases accompany oophagy. Egg-eating species within the marine-elapid genera *Aipysurus* and *Emydocephalus* consume fish eggs, prey that neither flee nor require chemical incapacitation. In *Aipysurus eydouxii*, the dietary transition has been shown to be associated with reduced venom toxicity, altered toxin expression, molecular degeneration, gland atrophy, and modification of the feeding apparatus [48,74,75]. The reduction is therefore mosaic rather than all-or-none: recognisable molecular or anatomical elements can persist after their original role in rapid prey subjugation has become ecologically redundant.

The same ecological route is repeated on land. Species of the African Colubroidea genus *Dasypeltis* swallow birds’ eggs whole and use enlarged, anteroventrally directed vertebral hypapophyses to rupture the shell within the oesophagus [131,132,133]. Several species within the Australian Elapidae genus *Brachyurophis*, treated within *Simoselaps* in much of the earlier literature, are specialised predators of reptile eggs [134,135]. Comparative reconstruction indicates that specialised oophagy in *Dasypeltis* and the lineage now recognised as *Brachyurophis semifasciatus* arose from ancestors that consumed the corresponding adult prey [136]. Both lineages are nested within Caenophidia, and *Brachyurophis* is nested within Elapidae [137,138]. In lineages reconstructed as ancestrally venomous, their convergence with egg-eating sea snakes is therefore consistent with the hypothesis that shifts to eggs relax selection on venom-mediated prey incapacitation and can be followed by secondary reduction, although not necessarily complete loss, of the venom system [48,49,74,75].

Mechanical prey restraint provides a second route. Rat snakes of the genus *Pantherophis* rely principally on constriction, and the atrophied venom-gland condition described in this lineage parallels the derived, mucus-dominant oral systems of boas and pythons [48,64]. These are independent mechanical solutions to the same functional problem: when rapid constriction reliably subdues prey, the fitness benefit of investing in chemical immobilisation can fall. Selection may then favour retention of lubrication for prey transport while allowing secretory tissue, toxin expression, or specialised delivery structures to regress.

File snakes (*Acrochordus*) provide a related but phylogenetically distinct comparison. They are caenophidians outside Colubroidea and use highly specialised aquatic mechanics (including rough, loose skin and body coiling) to grip slippery fish prey [139]. Their putative departure from a venom apparatus is compatible with reduced reliance on chemical subjugation, but the polarity is less secure than in losses nested within demonstrably venomous colubroid clades [49]. *Acrochordus* should therefore be treated as a candidate case of reduction, not as a confirmed loss or as a categorical proxy for the primitive caenophidian condition.

Reduced reliance on venom need not involve either eggs or constriction. Large, active colubrids such as *Drymarchon* and *Ptyas* species may rely heavily on mechanical prey handling. *Drymarchon* use high-pressure bites, prolonged jaw engagement, and muscular chewing, whereas *Ptyas* have been observed maintaining oral restraint during extended struggles and ingesting live snake prey [140,141,142,143,144,145]. Their behaviour suggests reduced toxin investment, but mechanical performance alone cannot establish loss: a lineage may retain a low-output secretion used only for difficult prey, defence, or another context [48,49]. As histological examinations to ascertain the relative state of the venom glands in these lineages have yet to be undertaken, these taxa are therefore better interpreted as candidates for secondary reduction than as demonstrated venom-free states.

Together, these cases show that venom-system loss is ecologically plausible, phylogenetically repeated, and capable of proceeding at different rates across glands, teeth, toxin genes, and behaviour. Ancestral degeneration predicts retained derived toxin architecture, lineage-specific disabling mutations or regulatory downshifts, and remnants of secretion in phylogenetically coherent positions [64,74,75,129,146]. While they do not demonstrate that functional venom arose at the root of Serpentes or before Caenophidia, and they do not convert ambiguous molecular remnants into evidence of current venom function, their historical implication is important: a weak or absent apparatus at a terminal taxon cannot automatically be coded as primitive absence. Evidence-weighted reconstruction should distinguish documented secondary loss, plausible reduction, uncertain state, and adequately sampled absence, while allowing both false-negative and false-positive classifications. The caenophidian examples therefore keep earlier-origin models viable and weaken categorical absence coding, but do not establish that an earlier model is better supported without a formal comparative reconstruction.

### 3.8. Scolecophidia Are Early-Diverging, Not Ancestral Proxies

Scolecophidia require the same evolutionary caution as constrictors, but for a different reason. Their early phylogenetic divergence does not imply retention of an unmodified ancestral phenotype. They possess highly modified fossorial skulls and restricted gape associated with specialised exploitation of ants, termites, and particularly their eggs, larvae, and pupae [49]. These relatively small and often immobile prey can relax selection on chemical subjugation, making reduction in an ancestral venom function biologically plausible, as seen for egg-eating sea snakes [74,75] and egg-eating colubrid snakes [48]. A negative venom assessment in a living scolecophidian would describe an extant state in a highly derived lineage, not the ancestral snake condition; whether that state represents secondary loss would remain to be reconstructed.

Available anatomy also prevents categorical coding of the entire lineage as primitively nonvenomous. Descriptions of typhlopid and anomalepidid snakes include large, predominantly serous glands beneath the eye, in some cases with a single duct and a secretion-filled storage lumen, that may be homologous to the venom glands of alethinophidian snakes [49]. These observations do not establish functional venom, and the glands and released secretions remain unsampled across Scolecophidia. They do establish that the relevant tip state is uncertain. Positive functional evidence would push the inferred origin towards the root of Serpentes, whereas failure to find venom in one or several highly specialised living taxa would not, by itself, exclude ancestral venom. Figure 3 summarises why the 107 blind-snake tips (concentrated in two phylogenetically correlated clades and coded nonvenomous in all four published schemes) should not be interpreted as 107 independent observations of the ancestral snake state, while separating the published inference from sensitivity treatments that remain to be analysed.

### 3.9. Caenophidian Anatomy Is an Elaboration, Not Necessarily the Starting Point

Caenophidians transformed the upper-jaw component of the oral secretory system. The gland became more sharply differentiated and, in many lineages, encapsulated; ducts established a close relationship with maxillary teeth; compressor musculature increased delivery pressure; and the dentition diversified into posteriorly enlarged or grooved teeth, fixed anterior fangs, and long rotating tubular fangs [48,52]. Developmental and comparative anatomical evidence indicates continuity among caenophidian rear-fanged and front-fanged systems: specialised fangs arise from a posterior dental field, and changes in maxillary development and growth reposition them [53]. The spectacular endoglyphous apparatuses are therefore repeated modifications of inherited developmental and secretory substrates, not wholly new inventions each time.

Those anatomical transitions improve metering, penetration, speed, and reliability. They do not date the first moment at which an oral secretion perturbed prey or predator physiology. Rear-fanged, fixed-front-fanged, and hinge-fanged snakes can share an older venom function while independently improving tooth position, groove closure, gland storage, or muscular compression [48,53]. Conversely, a lineage can reduce its apparatus while retaining some molecular components. The observation that complex delivery architectures evolved repeatedly is therefore evidence of selection on delivery efficiency; it is not evidence that the underlying venom function arose de novo with each architecture.

The same temporal logic in lizards of a mixed seromucous gland preceding segregation of mucous and protein regions into distinct glands [51] supplies a testable hypothesis within snakes. If enlargement, encapsulation, ducting, and associated dental changes primarily improved the delivery of toxic proteins, they represent elaboration after a secretion had acquired a fitness-relevant effect. A distributed serous or seromucous oral system, such as that retained in *Cylindrophis*, is therefore a candidate antecedent. Its status depends on natural secretion, wound delivery, realistic dose, and a secretion-specific fitness benefit. Wüster, Modahl, and Arbuckle’s species-level coding places the relevant non-caenophidian tips in the nonvenomous state across all four schemes [14]; it therefore does not test this alternative characterisation. This leaves the alternative open for future evidence-audited recoding and reanalysis.

### 3.10. Extend Evolutionary Tinkering One Node Deeper

Fry and Wüster used toxin phylogenies and the distribution of venom-delivery characters to argue for an early origin within advanced snakes followed by repeated recruitment, diversification, loss, and specialisation [146]. Their central conceptual point was that evolution assembles an arsenal by modifying inherited components: independent elaboration of fangs does not require independent origins of the secretion delivered by them. The current proposal in this manuscript extends that reasoning one node deeper. If early alethinophidians possessed distributed protein-secreting oral glands, derived oral isoforms, and a low-output toxic secretion, then the caenophidian gland represents concentration and elaboration of the maxillary region rather than the first appearance of venom.

This extension does not require acceptance of every toxin homology proposed in early Toxicofera studies [10]. The original hypothesis of an ancient shared oral venom system in snakes and anguimorph lizards stimulated the comparative work but also bundled molecular availability, tissue expression, secretion, function, and apparatus into an inference that later transcriptomic studies correctly challenged [18]. The within-snake hypothesis can be evaluated more narrowly and with stricter evidence: developmental continuity of oral gland fields, derived paralogues rather than family labels, gland-specific expression, secreted mature products, realistic physiological activity, and natural delivery.

A remaining anatomical uncertainty is the homology of specific *Cylindrophis* compartments with the specialised caenophidian gland. Similar position and shared oral expression make continuity plausible, but developmental fate mapping and cell-type comparisons are required to show whether the serous maxillary tissue is directly homologous to the caenophidian venom-gland lineage, whether several circumlabial fields contributed, or whether some protein-rich compartments arose independently. An early functional secretion does not require every gland compartment to be one-to-one homologous; it does require evidence that at least part of the ancestral oral system was inherited and elaborated rather than reinvented.

### 3.11. Evidentiary Status of an Earlier Within-Snake Origin

The confidence attached to each node must remain explicit. An origin before Colubroidea is well supported because caenophidian venom glands and toxin systems are homologous across lineages whose fang architectures were elaborated later and repeatedly. An origin before Caenophidia remains a biologically explicit and viable alternative because anatomical, molecular, secretory, and historical observations outside Caenophidia are compatible with it, but those layers have not been united in one phylogenetically critical taxon or evaluated through evidence-audited alternative coding. A retained low-output system near the base of Alethinophidia is therefore a priority hypothesis for testing. An origin at the root of Serpentes remains data deficient because direct functional evidence from Scolecophidia and several other early lineages is lacking. It is not contradicted by their present condition: the derived morphology and ecology of living Scolecophidia prevent apparent venom absence from polarising the ancestral state by itself.

Three caveats prevent a stronger conclusion. First, the reports for *Eryx* and uropeltids are positive but methodologically antiquated and must be replicated with anatomically identified secretions, controlled doses, and relevant assays. Secondly, *Cylindrophis* itself still lacks direct secretome-scale proteomic confirmation and modern tests of physiological and ecological function; proteomics from the comparable but phylogenetically separate *Aspidites* cannot fill that gap. Thirdly, negative evidence from Scolecophidia or any other lineage should be accepted only after adequate sampling of the appropriate maxillary, mandibular, and rictal tissues, at relevant physiological states, with sensitive proteomics and explicit power to detect low-output secretion.

The evidentiary basis for an earlier origin would be strengthened most by a single integrative *Cylindrophis* study that links gland cell types, spatial expression, mature secreted isoforms, dose–response activity, and natural bite performance. Comparable positive evidence from additional uropeltoids would strengthen the basis for testing a deeper placement. Negative findings in specialised scolecophidian lineages, however, should not be treated as a decisive test of the root state. Ancestral-state analyses should code each evidentiary layer separately, represent inadequately sampled taxa as unknown, allow realistic rates of reduction and loss, and model false-positive and false-negative classification. The competing within-lineage histories for Anguimorpha and Serpentes (distinguishing published reconstruction, observed anatomical change, and earlier functional hypotheses requiring testing) are synthesised in Figure 4.

## 4. Linking Lizards and Snakes: One Origin, Two Origins, or Insufficient Data?

### 4.1. Wüster, Modahl, and Arbuckle’s Lineage-Level Reconstructions Warrant Broader Testing: The Deeper Functional Homology Remains Unresolved

Wüster, Modahl, and Arbuckle’s inference that squamate venom does not have a single functional origin is internally robust to the four coding schemes they tested [14]. It is based on species-level data, explicit unknown states, and a precursor model that permits loss. The present review therefore does not supply a categorical correction to that reconstruction. It instead identifies biological alternatives that remain outside the tested model and coding set: a finer division of precursor states, bidirectional, evidence-calibrated observation error, additional anguimorph taxa, and alternative non-caenophidian snake classifications. Within Anguimorpha, glandular, molecular, proteomic, and pharmacological evidence warrants testing an earlier functional origin followed by parallel elaboration. Within snakes, evidence from non-caenophidian oral glands warrants investigation of alternative coding to the inferred Caenophidia-only origin [14]. Because the present review does not perform that reconstruction, it does not rank the alternatives; they are presented as testable hypotheses rather than established lineage-level corrections.

### 4.2. Single Versus Multiple Origins of Venom in Squamate Reptiles

#### 4.2.1. Define the Competing Hypotheses Precisely

The alternatives must be defined at the same biological level (Table 2). ‘One origin’ can refer to the origin of a protein-secreting oral field, the first oral expression of a physiological gene, the first specialised toxin isoform, the first secretion that improved fitness through toxicity, or the first elaborate delivery apparatus. These are not equivalent. The present comparison concerns functional venom: a secretion delivered through an inflicted wound that perturbs another organism in a fitness-relevant interaction. Homology of glands or regulatory substrates is therefore evidence about evolutionary capacity and continuity, but it is not, by itself, evidence that the ancestral secretion had crossed the functional threshold.

Table 2 separates two hypotheses that are often collapsed under the phrase ‘multiple origins’. Under the shared-precursor model, the deep ancestor already possessed homologous protein-secreting oral tissues and accessible molecular substrates, while anguimorphs and snakes independently crossed the functional threshold. A more strongly convergent model would place less specialised continuity in that oral context. These distinctions make different predictions about secretory-cell homology, developmental patterning, spatial regulatory programmes, and derived toxin isoforms. Importantly, the latter extreme should not be attributed to Wüster, Modahl, and Arbuckle: their best-fitting model explicitly contains an incipient precursor state. Their inference concerns the number and placement of precursor and functional transitions, not origins from biological blank slates.

#### 4.2.2. Evidence Consistent with a Single Shared Functional Origin

Anatomical continuity cannot alone establish ancestral toxicity. Protein-rich saliva can serve lubrication, digestion, antimicrobial defence, wound maintenance, or prey adhesion, and the same gland can be multifunctional. Its relevance is predictive rather than dispositive. A single-functional-origin model predicts homologous secretory cell states, developmental patterning, and derived regulatory or molecular innovations across oral positions, even where mature glands differ dramatically. A two-functionalisation model can share the broad anatomical and regulatory substrate but predicts independent specialisation near each crossing of the functional threshold. These alternatives should be compared through their biological predictions and fitted transition histories; the number of apparently required changes is not, by itself, statistical support.

#### 4.2.3. Reduction and Loss Make Living Absences Poor Temporal Markers

A shared origin does not predict an obvious venom system in every descendant. Venom traits are repeatedly reduced, deleted, repurposed, or replaced when ecology changes [20]. Gene-tree reconstructions show toxin lineages returning to physiological expression or being lost from particular descendants, and genomic work in *Crotalus* species (rattlesnakes) demonstrates recent deletion of ancient toxin genes [73]. Comparative analyses across tetrapods likewise show that venom can evolve and disappear rapidly relative to deep lineage age [71].

This dynamic history is particularly relevant to lineages whose feeding ecology supplies an alternative to chemical subjugation. Powerful constriction can relax selection on venom in boas and pythons, favouring low toxin abundance and a greater mucous contribution to lubricating large prey [64]; the resulting mucus-dominated anatomy is derived rather than a retained pre-venom condition. Scolecophidia are likewise highly transformed for fossoriality and specialised feeding on ants, termites, and their brood [49]. Neither group is therefore an appropriate proxy for a primitively nonvenomous snake ancestor. Apparent absence in these living lineages is compatible with secondary reduction and cannot, without additional evidence, be used against either an origin at the root of Serpentes or a shared lizard-snake venom system. Strong mechanical processing, dietary shifts, or defensive changes could generate comparable reductions in lizards and iguanians.

#### 4.2.4. Evidence Consistent with Two Independent Functional Origins

##### Different Anatomical Emphases May Reflect Separate Functionalisation

The strongest anatomical argument for two functional origins is not that lizards and snakes possess completely unrelated oral tissues, but that selection concentrated on different parts of a homologous field. Anguimorph specialisation is predominantly mandibular, whereas caenophidian specialisation is predominantly maxillary. If the ancestral condition was a distributed, low-output seromucous system with no toxic ecological role, then the two lineages may independently have intensified different components as toxic delivery systems. Under this model, shared anatomy explains why similar transitions were accessible, while distinct sites of elaboration record separate crossings of the functional threshold. 

An argument against this is the demonstration that some anguimorph lizards regain maxillary glands [51] and that the *C. ruffus* snake transcribed identical toxin types in both the mandibular and maxillary regions [64]. The two-function hypothesis becomes compelling only if comparative cell biology shows that toxin-secreting states arose independently in the mandibular lizard and maxillary snake components, or if orthology-resolved toxin histories consistently place functional recruitment after the two lineages diverged. Mature gland position supplies a prediction; it is not by itself the answer.

Crucially, the two-state inference must be separated from the later evolution of encapsulation, ducts, compressor muscles, and dental specialisation. Different mature architectures prove different elaboration histories, not different origins of the underlying secretion.

##### Ancient Physiological Substrates Can Be Co-Opted Repeatedly

Most venom proteins were not invented de novo. They descend from physiological proteins involved in haemostasis, inflammation, signalling, digestion, immunity, or tissue maintenance [1,15]. Broad expression of homologues across oral and non-oral tissues means that similar toxin families can be recruited independently, particularly where the same recipient pathways offer vulnerable pharmacological targets. Comparative transcriptomic critiques demonstrated that several early claims of Toxicofera-wide toxin recruitment relied on sparse non-toxicoferan sampling, inadequate non-oral controls, or failure to distinguish physiological orthologues from toxin paralogues [16,17,18]. Those critiques materially weakened the inference that every shared family records ancestral venom. Convergence must therefore remain an explicit alternative to deep functional homology: comparative transcriptomic analysis across Metazoa has found convergent venom-gland expression involving shared cellular and secretory functions, showing that similarity at the gland-transcriptome level can arise independently and must be tested with finer orthology, cell-type, and regulatory evidence [147].

A conserved amniote ‘metavenom’ regulatory network strengthens the precursor model while remaining ambiguous about function. Barua and Mikheyev identified an ancient gene-regulatory network associated with secretory protein homeostasis and found conservation extending beyond venomous squamates [19]. Such a network can preserve the molecular competence from which oral venom systems evolve, but its antiquity means that it cannot uniquely diagnose functional venom at the root of Toxicofera. It is compatible with one inherited toxic secretion, yet it also makes repeated co-option easier because both lineages begin with a similar regulatory substrate.

##### The Genomic Critiques Set the Correct Evidentiary Threshold

The principal genomic critiques should not be dismissed as semantic disputes over the word ‘toxin’. Their central point is correct: a familiar venom-family name attached to a transcript does not establish toxin recruitment, secretion, or ecological use. Low-level expression of homologues must be distinguished from oral enrichment; gene duplication from orthology; translated secretion from transcription; realistic target activity from generic biochemical effects; and an effect at a delivered dose from an effect after artificial high-dose injection. The burden is especially high at the lizard–snake split because a false assignment of one cross-lineage homology can shift the inferred origin by tens of millions of years.

Accepting that threshold does not erase the positive evidence reviewed in the preceding sections. Derived sequence features in early snake three-finger toxins, protein-level confirmation in a pythonid, unusual precursor processing within anguimorphs, secreted kallikreins and other active components in specialised lizards, and pharmacological innovations such as celestoxin are not equivalent to unquantified housekeeping transcripts. Rather, the critiques specify why those observations must be connected across lineages by synteny, cell type, secretion, and ecological performance before one deep functional origin can be claimed. At present, the required bridge has not been built.

#### 4.2.5. Separate Functional Origins Can Still Share an Inherited Oral Substrate

Evidence for separate functional origins does not require lizard and snake venoms to have arisen from wholly unrelated starting points. Homologous oral epithelia, a conserved secretory network, distributed serous compartments, and recurring physiological substrates probably predated both mature systems. This is also compatible with the precursor framework fitted by Wüster, Modahl, and Arbuckle. The central alternatives are therefore one inherited functional secretion versus two crossings of the functional threshold from a shared nonvenomous substrate. Current anatomy and molecular biology generate discriminating predictions for those histories, but event counting or an appeal to biological economy cannot establish their relative likelihood.

#### 4.2.6. Phylogeny Changes the Ancestral Node Being Tested

Any statement about a shared origin must specify the phylogeny. Toxicofera is the clade comprising Serpentes, Anguimorpha, and Iguania [148], but the branching order among those three lineages has remained difficult to resolve. The widely favoured phylogenomic topology places Serpentes as sister to an Iguania–Anguimorpha clade [125,148,149]. Under that topology, the most recent common ancestor of venomous anguimorphs and snakes is the root of Toxicofera and is also ancestral to Iguania. A single shared functional origin would therefore imply that the toxicoferan ancestor was venomous before the iguanian split, followed by strong reduction or loss in much of Iguania. The principal topology discussed here has been recovered in large multilocus and integrated morphological–molecular datasets, although analyses of thousands of nuclear loci have also emphasised both the placement of snakes and the extent of conflict among squamate relationships [150,151,152].

The internal toxicoferan topology should not be presented as settled, however. Genome-wide analyses reveal substantial gene-tree conflict, with support distributed among the three possible arrangements [153]. A likelihood-based comparison across genomic datasets recovered unexpectedly broad support for a Serpentes–Iguania grouping, and analysis incorporating the genome of the relict earless monitor *Lanthanotus borneensis* found Toxicofera itself supported by the plurality of gene trees but the three internal arrangements nearly evenly represented, with only a slight plurality for Iguania–Anguimorpha [154]. Such conflict may reflect short internodes, incomplete lineage sorting, model misspecification, or other sources of discordance.

Under an alternative topology that directly pairs Anguimorpha and Serpentes, their shared functional origin could be placed on the common stem after Iguania had diverged. That history would not require an iguanian venomous ancestor. Under a Serpentes–Iguania topology, by contrast, a venom system restricted ancestrally to Anguimorpha plus Serpentes would demand either convergence or a more complex sequence of gains and losses. The biological conclusion is therefore conditional on both the character model and the species tree. Ancestral-state analyses should integrate plausible topologies rather than map a binary character onto a single fixed tree and treat node identity as unambiguous.

#### 4.2.7. Iguania Is the Decisive Third Lineage

Under the commonly recovered Serpentes sister to Iguania–Anguimorpha topology, Iguania is not a peripheral test. It is a descendant of the exact ancestral node at which a single lizard–snake origin would be placed. Iguanians possess oral serous or mixed seromucous glands in both jaws, and early comparative work reported transcripts assigned to several toxin-related families in *Pogona* and other iguanian material [10,64]. These findings support a homologous oral secretory substrate and demonstrate molecular availability. They do not yet provide equivalent evidence for a released toxic secretion, a realistic dose, and an ecological benefit.

The iguanian prediction is consequently sharp. If the toxicoferan ancestor possessed functional venom, living iguanians should retain some combination of homologous toxin isoforms, oral cell states, regulatory elements, secreted products, or genomic remnants of reduction, even if modern ecological use has largely disappeared, as investigated previously [64]. If the ancestor possessed only a nonvenomous secretory precursor, iguanians may share the anatomy and regulatory network without showing toxin-specific enrichment, secretion, or function. Modern matched analyses of upper- and lower-jaw glands, saliva collected without tissue disruption, quantitative proteomics, prey-relevant pharmacology, and natural-bite performance are therefore capable of discriminating the models. Until that work is done, coding Iguania categorically as either venomous or nonvenomous risks replacing the test with an assumption.

#### 4.2.8. The Weak Toxicofera Hypothesis Is Not One Origin of Venom

A semantic distinction is essential. The ‘strong’ Toxicofera hypothesis proposes functional venom in the toxicoferan ancestor [14]. A ‘weak’ version proposes an ancestral nonvenomous precursor, such as protein-secreting oral glands, an accessible regulatory programme, or molecular substrates, from which functional venom arose later [14]. The weak hypothesis can therefore accommodate one or more origins of venom function. It describes one origin or inheritance of the substrate, not necessarily one origin of the trait being tested. Wüster, Modahl, and Arbuckle correctly preserve this distinction. Their inference against one functional origin is the conditional result of their fitted transition model and tip codings [14]; it should not be represented as a claim that lizard and snake venoms arose from unrelated biological blank slates.

This distinction also exposes a limitation of representing the history with one hidden ‘precursor’ state. A plausible path may include several separable transitions: broadly distributed seromucous glands, oral enrichment of a physiological paralogue, translation and release, recipient activity at a delivered dose, a measurable ecological benefit, and later architectural elaboration. Different lineages can retain different subsets of those states, and reductions can run in the reverse direction. Collapsing the pathway into precursor versus venom makes estimation tractable, but the inferred transition then depends on where investigators place a single threshold across a biologically graded system. A multistate or hidden-rate framework that represents bidirectional observation error, unknown tips, and realistic loss may infer a different number and placement of functional gains without changing any raw observation.

#### 4.2.9. Why the Deepest Conclusion Remains Data Deficient

The evidence is uneven across lineages and incomplete in both positive and negative directions. Within Anguimorpha, the Anguoidea bridge taxa connect simple mandibular secretory compartments to specialised *Heloderma* and varanoid systems through derived molecules, translated products, and physiological activity, but these layers do not consistently establish expelled secretion, natural delivery, realistic dose, and fitness benefit in the same bridge taxon. Within Serpentes, non-caenophidian anatomy, coordinated oral expression, pythonid secretion proteomics, and historical functional observations make an origin below Caenophidia worth testing, but historical gland attribution and dosing, as well as modern natural-delivery effects, remain uncertain. Across the lizard-snake divide, however, similar glands and recurrent toxin families do not demonstrate that the same ancestral secretion was ecologically venomous. The molecular substrate may be homologous while the functional threshold was crossed once or twice. The present evidence identifies discriminating characters and shows that categorical absence coding is insufficiently secure for several poorly investigated taxa, but it does not select among the competing histories.

The converse inference is equally invalid. Failure to demonstrate a functional secretion in a deep ancestor cannot establish two origins because the ancestor cannot be assayed directly, many living proxy taxa are poorly sampled, and even rigorously demonstrated absence in a derived descendant does not identify the ancestral state without an explicit model of loss. In snakes, negative results from derived mucus-specialised boids or pythonids and ecologically specialised derived Scolecophidia would remain compatible with secondary reduction. Negative evidence is meaningful only after anatomically appropriate glands and released secretions have been examined with methods capable of detecting low-output systems and the direction of anatomical change has been reconstructed. A phylogeny populated with assumed zeros will inevitably fragment positive tips into gains; a phylogeny populated indiscriminately with positives will push function too deep. The appropriate response is to represent uncertainty in both directions and design observations that change the relative likelihood of the alternatives.

#### 4.2.10. A Discriminating Comparative Programme

The decisive programme must compare homologous biological levels across the three toxicoferan lineages. Anatomically, it should map serous and mucous cell types, storage lumina, ducts, innervation, developmental origin, and their relationship to the tooth row in maxillary, mandibular, and rictal regions. Molecularly, it should use chromosome-scale synteny and densely sampled gene trees to distinguish physiological orthologues, toxin paralogues, duplications, and losses; precursor processing and derived structural features should be tested explicitly. Regulatory comparisons should combine single-cell and spatial transcriptomics with chromatin accessibility and enhancer analyses to determine whether equivalent oral cell states use homologous programmes.

Secretion and function must then be tested rather than inferred from transcripts. Material should be collected from anatomically identified glands without destructive contamination, quantified by absolute and targeted proteomics, and related to the amount entering a wound during natural biting and holding. Candidate effects should be assayed at those concentrations against ecologically relevant vertebrate and invertebrate prey, predators, or competitors, with comparisons to physiological homologues and non-oral secretions. The crucial ecological outcomes are changes in immobilisation time, capture success, prey escape, handling injury, predator deterrence, or other fitness-relevant performance, rather than simply activity in a permissive biochemical assay.

Those outcomes are not self-interpreting. Natural-history observations should determine which recipients, behaviours, seasons, and performance variables are biologically relevant, but causal attribution requires a secretion-specific counterfactual. The experimental manipulation must change toxin exposure without changing the mechanical wound, restraint, handling, motivation, or recipient condition; otherwise, the effect remains attributable only to the composite bite. No single design will suit every lineage, so duct occlusion, depletion-and-recovery, selective neutralisation, and standardised bite-proxy approaches should be validated taxon by taxon. This is the methodological step missing when ‘ecological evidence’ is invoked as a future solution without specifying what observation would discriminate venom from biting itself.

Tip evidence is phylogenetically informative only to the extent that the study design bounds misclassification. A negative result is informative when the study can exclude a pre-defined effect small enough to matter to fitness under the tested conditions; failure to reach statistical significance, observe dramatic impairment, or encounter the relevant behaviour in the field cannot meet that standard. Positive evidence requires corresponding scrutiny: a historical experiment with uncertain gland identification or dose, protein detected only in gland extract, a toxin-family transcript without demonstrated secretion, or an effect observed only at a high artificial dose should increase, but not fix, the probability of functional venom. For low-output bridge taxa, most present datasets should therefore yield graded likelihoods or unknown states rather than forced positive or nonvenomous classifications. A positive controlled performance effect need not be large or indispensable to every interaction; a reproducible probabilistic advantage at realistic delivery is sufficient to support present ecological function.

Taxonomic sampling should prioritise lineages that alter ancestral-state placement rather than those with the largest or easiest glands. Within snakes, this includes multiple scolecophidian families, *Cylindrophis*, uropeltids, *Eryx*, and multiple boid and pythonid constrictors. Scolecophidian sampling should test the reported serous glands across distinct lineages and feeding specialisations rather than extrapolating from one highly modified species; constrictor sampling should distinguish derived mucus-dominated tissues from retained serous compartments and genomic remnants of reduction. Within anguimorphs, sampling should include Anguoidea such as *Abronia*, *Celestus*, *Gerrhonotus*, and related genera, varanoids such as *Lanthanotus* and additional *Varanus* species. Across the deeper node, broad sampling of Iguania is indispensable. Replicates should encompass diet, age, sex, season, and recent feeding because a negative sample from a conditionally active gland is not a reliable species-level absence.

Finally, ancestral reconstruction should follow the biology. Tip states should be multivariate or explicitly uncertain, distinguishing molecular availability, toxin-specialised expression, release, realistic activity, ecological benefit, and delivery elaboration. Observation models should allow evidence-calibrated false-negative and false-positive classifications; transition models should accommodate reduction and loss; species-level resolution should be retained, as in the Wüster, Modahl, and Arbuckle analysis [14]; and plausible toxicoferan topologies should be integrated. If such analyses become possible, support for one versus several gains should be quantified through ancestral-state probabilities, stochastic mappings, and event-count distributions rather than inferred from apparent parsimony alone. The present narrative review does not perform this comparison and therefore does not rank the competing histories.

## 5. Conclusions

The strongest current conclusion is conditional and layered. The Wüster, Modahl, and Arbuckle reconstruction is internally robust to the four coding schemes tested and supports separate functional gains under its species-level data, unknown-state treatment, loss-permitting transition model, and single hidden precursor [14]. The anatomical, molecular, proteomic, and pharmacological evidence reviewed here identifies biologically explicit alternative characterisations, particularly for non-caenophidian snakes and graded precursor states, but does not test them quantitatively against the published model [14]. Earlier functional origins within Anguimorpha and Serpentes therefore remain viable hypotheses, and categorical absence coding in several critical, poorly investigated taxa is not sufficiently secure to exclude them. Because no alternative reconstruction is performed here, the review does not estimate their relative support.

The architectural sequence remains relevant to these alternative hypotheses. If enlargement, encapsulation, storage, and ducting primarily improved the delivery of toxic proteins, they are more plausibly elaborations of an already beneficial secretion than markers of its first origin. Anguoidea such as *Abronia*, *Celestus*, and *Gerrhonotus*, and non-caenophidian snakes such as *Cylindrophis*, provide important tests of that sequence. Wüster, Modahl, and Arbuckle also code selected simple-gland anguimorphs as venomous or unknown rather than recognising only elaborate endpoints [14]. Thus, anatomy challenges the equation of simple glands with confirmed absence, but it does not establish an earlier functional state or make that history better supported.

Derived absences are as important as derived elaborations. The predominantly mucous systems of boas and pythons are plausibly secondary specialisations associated with constriction and large-prey handling [64], while Scolecophidia are highly modified for fossoriality and feeding on social-insect brood [49]. Neither condition, by itself, establishes a primitively nonvenomous snake ancestor. Even confirmed modern absence would remain compatible with secondary reduction under an appropriate transition model. Conversely, compatibility with reduction is not evidence that reduction occurred. These taxa leave an earlier snake origin open for testing rather than confirming or falsifying it.

Natural history is essential evidence for identifying plausible recipients and present ecological functions. The repeated *Heloderma* diet studies, for example, materially favour defence as a major current role, but the seasonal and observational biases of these studies may obscure the role in predation. Natural history does not, by itself, identify the historical interaction in which a venom system originated, and absence of a conspicuous effect is weak negative evidence when observation and detection are poor. Conversely, a well-designed negative study of the anatomically appropriate gland, released secretion, natural dose, and relevant performance outcome should not automatically be recoded as unknown. Apparent positives also require graded treatment when gland identity, secretion, delivered dose, or a causal performance effect is uncertain. Ancestral-state analyses should distinguish rigorous negative evidence from inadequate investigation and strong positive evidence from suggestive or indirect observations.

At the deeper lizard–snake node, the evidence remains insufficient to distinguish decisively between one shared functional origin and two independent functionalisations of a homologous oral secretory precursor. Wüster, Modahl, and Arbuckle recover separate functional gains under the four tested codings [14]; the present review identifies biological reasons why earlier states remain viable and why some categorical absences warrant lower confidence. Because an evidence-audited alternative reconstruction is not available here, no claim is made that an earlier origin is better supported. The appropriate conclusion is data deficiency beyond the fitted model set, with uncertainty retained in both positive and negative tip classifications [14].

## Figures and Tables

**Figure 1:**
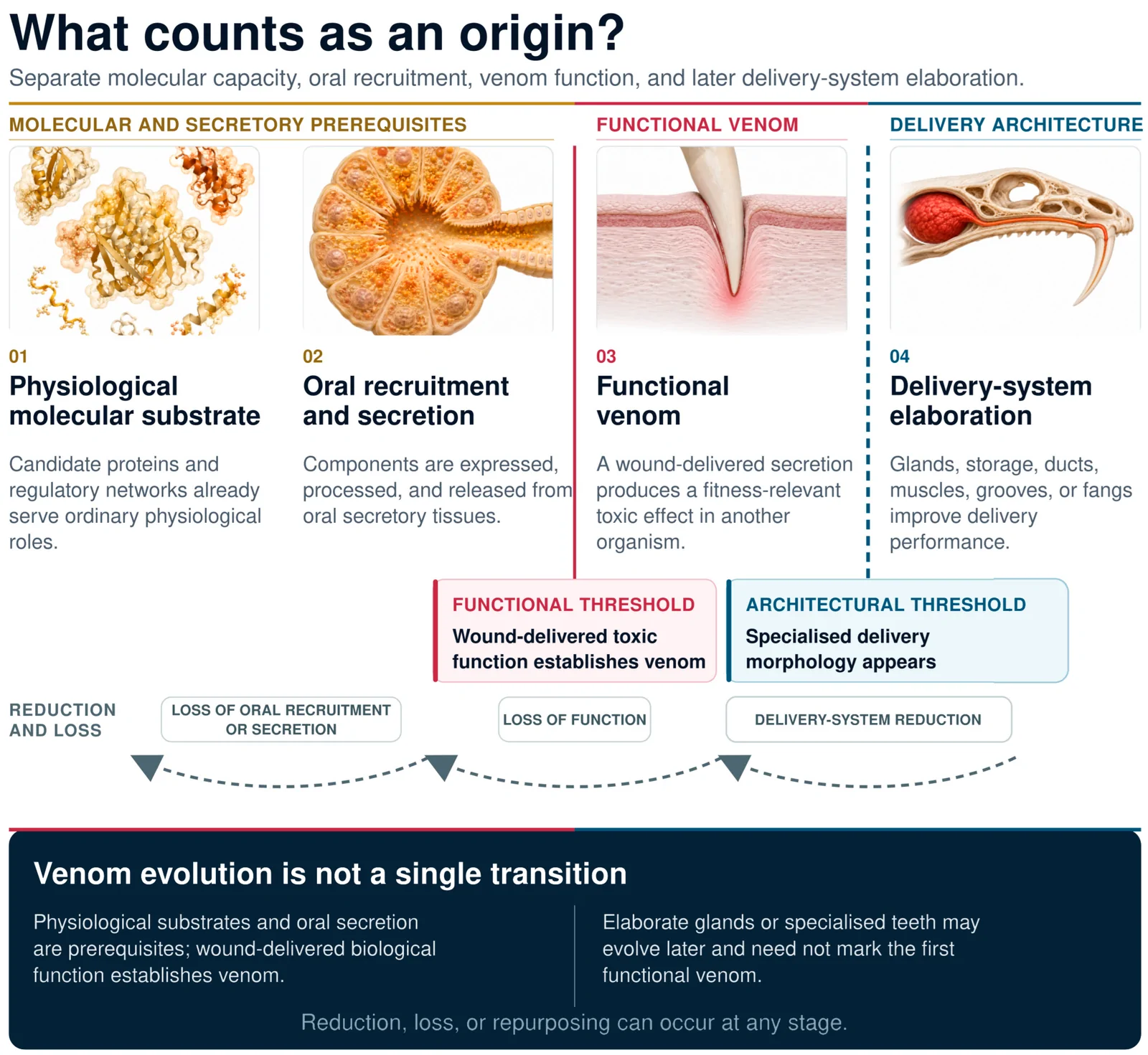
What counts as an origin? Conceptual separation of four stages in venom evolution: an ancestral physiological molecular substrate, recruitment and secretion through oral tissues, the emergence of functional venom, and subsequent elaboration of the delivery system. Candidate proteins and regulatory networks may therefore pre-date oral secretion, while oral expression alone does not demonstrate venom function. The functional threshold is crossed when a wound-delivered secretion produces a biologically and fitness-relevant toxic effect in another organism, but this does not require specialised glands or intricate delivery architecture. Specialised glands, storage compartments, ducts, musculature, dental grooves, or fangs may evolve later and consequently are not signatures of the earliest functional venom. Reverse pathways indicate that secretion, function, or delivery architecture may subsequently be reduced, lost, or repurposed. The stages are conceptual distinctions rather than an obligatory invariant sequence, and the illustrations are schematic and not to scale.

**Figure 2:**
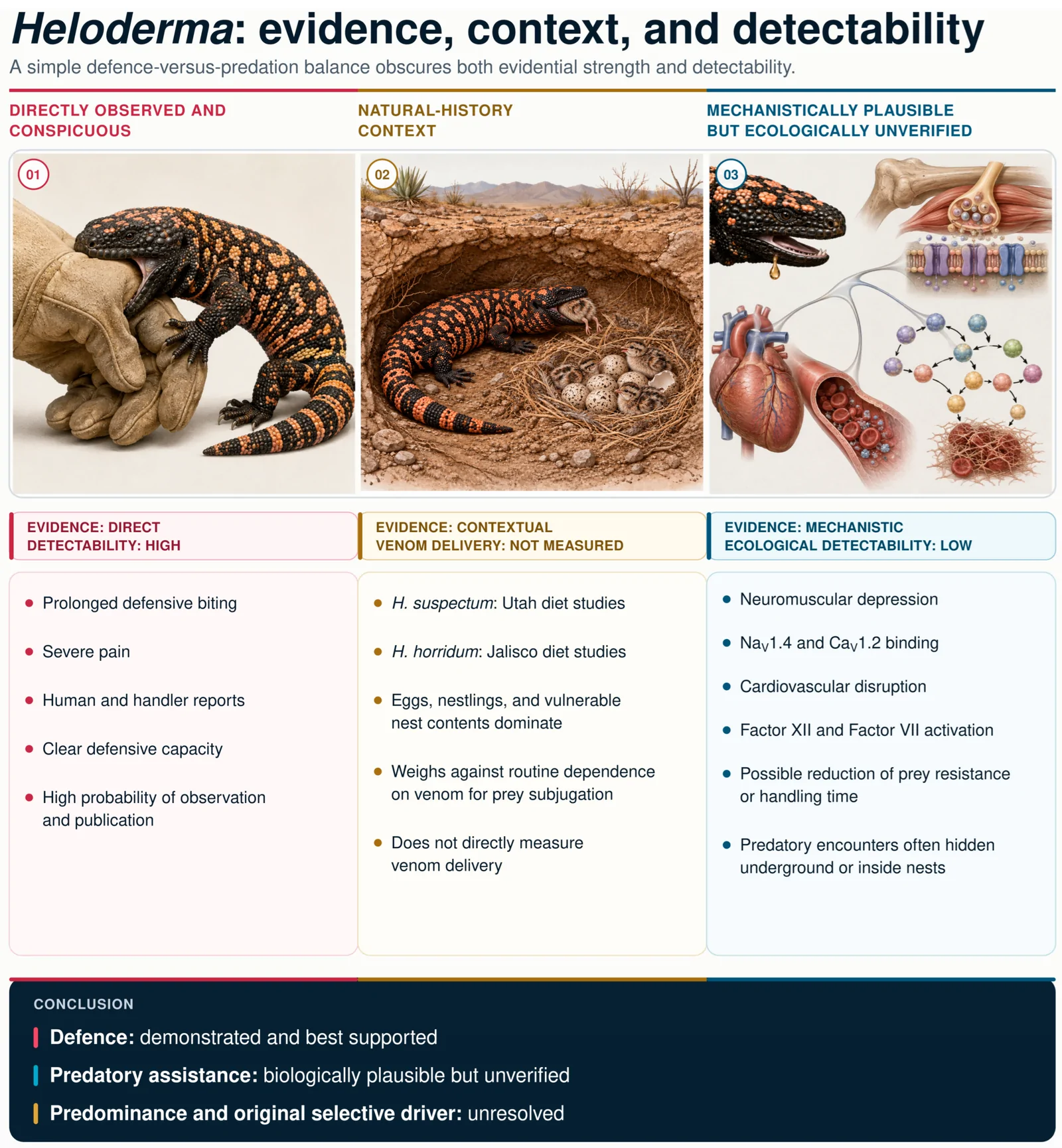
Evidence concerning defensive and predatory functions is divided according to its directness and probability of observation. Prolonged defensive biting, severe pain, and reports involving humans or animal handlers directly demonstrate defensive capacity and are both conspicuous and likely to be recorded. Natural-history studies of *H. suspectum* in Utah and *H. horridum* in Jalisco show diets dominated by eggs, nestlings, and other vulnerable nest contents, but these studies are biased due to the lizards being above ground during the bird breeding season. Predatory encounters occurring underground or within nests have low observational and publication probabilities. Neuromuscular depression, Na_V_1.4 and Ca_V_1.2 binding, cardiovascular disruption, and activation of blood clotting factors provide mechanistic grounds for possible predatory assistance, such reduced prey resistance or handling time for difficult prey such as rodents. Defence is therefore demonstrated and currently best supported, while predatory roles remains biologically plausible but ecologically unverified. Consequently the predominant function and original selective driver (defence versus predation) remain unresolved.

**Figure 3:**
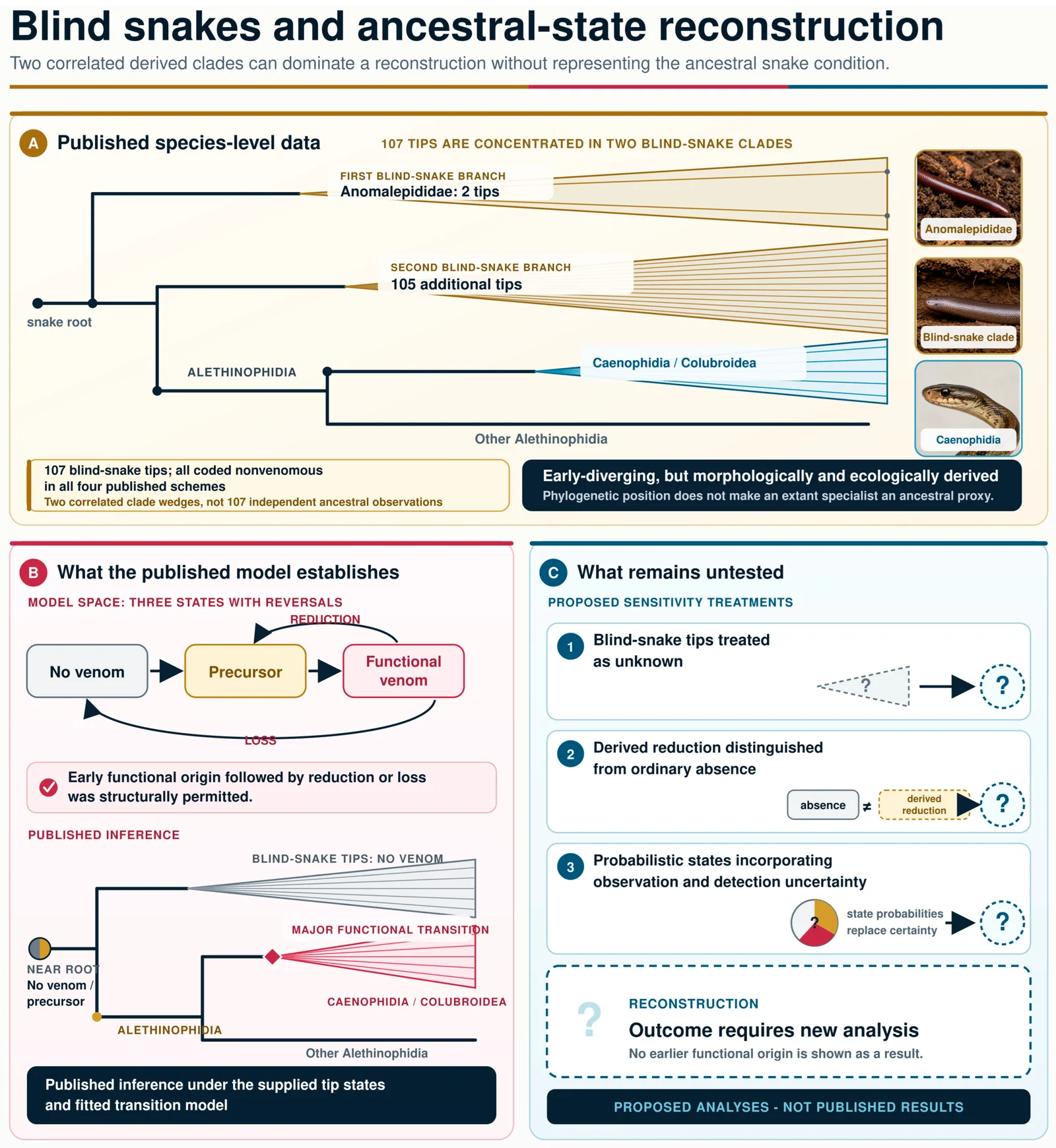
Blind snakes and ancestral-state reconstruction. (**A**) Representation of the published species-level dataset, in which 107 blind-snake tips were coded as nonvenomous under all four coding schemes. These tips are concentrated within two phylogenetically correlated clades (two anomalepidid tips and 105 additional blind-snake tips) and therefore do not constitute 107 independent observations of the ancestral snake condition. Although early-diverging, living blind snakes are morphologically and ecologically specialised and should not be treated as unspecialised proxies for the snake ancestor. (**B**) The published three-state model included no venom, precursor, and functional venom states and permitted reversals from functional venom to precursor or nonvenomous conditions. An early functional origin followed by reduction or loss was therefore structurally allowed. Nevertheless, under the supplied tip states and fitted transition model, the published reconstruction inferred a nonvenomous or precursor condition near the snake root and a major functional transition near Colubroidea. (**C**) Proposed sensitivity treatments include auditing the evidence for each blind-snake tip, using unknown states only when the evidence is genuinely uninformative, distinguishing derived reduction from ordinary absence, and assigning probabilistic states that incorporate both false-negative and false-positive classification. The question marks indicate that these treatments require new analyses; no earlier functional origin is presented as an inferred result. Taxon illustrations and phylogenies are schematic and not to scale. Reference to published data refers to Wüster, Modahl, and Arbuckle’s [14].

**Figure 4:**
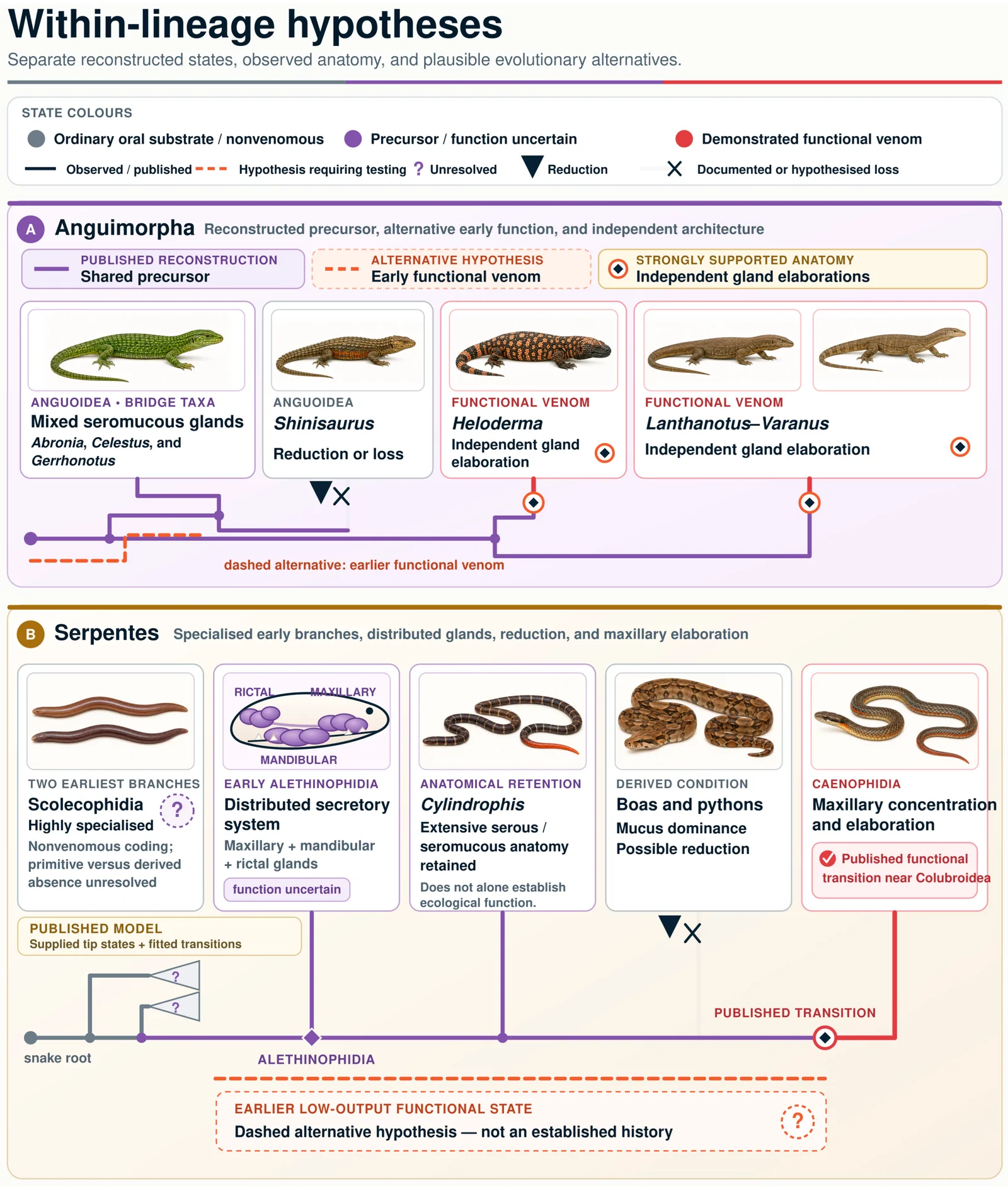
Within-lineage hypotheses for Anguimorpha and Serpentes. Grey denotes an ordinary oral secretory substrate or nonvenomous condition, purple denotes a precursor or functionally uncertain secretion, and orange-red denotes demonstrated functional venom. Solid paths represent observed features or published inferences, whereas dashed paths represent biological hypotheses requiring further testing. Question marks indicate unresolved states, downward arrows indicate reduction and fading, or crossed branches indicate documented or hypothesised loss. (**A**) In Anguimorpha, the published reconstruction supports a shared precursor. Mixed seromucous glands in Anguoidea, including the bridge taxa *Abronia*, *Celestus*, and *Gerrhonotus*, are shown alongside possible reduction or loss in *Shinisaurus*. Independent gland elaborations in *Heloderma* and the *Lanthanotus–Varanus* lineage are represented as strongly supported anatomical transitions. An early origin of functional venom is shown only as a dashed alternative hypothesis and not as the published reconstruction. (**B**) Scolecophidia are represented as two early-diverging but highly specialised branches whose nonvenomous coding cannot presently distinguish primitive absence from derived reduction. Early Alethinophidia are shown with a distributed maxillary–mandibular–rictal secretory system, with extensive serous or seromucous anatomy retained in *Cylindrophis*. Boas and pythons exhibit derived mucus dominance and possible reduction, whereas Caenophidia show maxillary concentration and elaboration. The solid orange-red transition near Colubroidea represents the published functional inference; the dashed earlier low-output functional state is an alternative requiring new analysis, not an established evolutionary history. Taxon illustrations and phylogenies are schematic and not to scale. Reference to published data refers to Wüster, Modahl, and Arbuckle’s [14].

**Table 1:** Comparative evidence bearing on an early anguimorph origin of functional venom.

Taxon or Lineage	Gland Evidence	Toxin or Secretion Evidence	Evolutionary Implication
*Abronia*	Robust mandibular glands formed from repeated mixed seromucous compartments along the tooth row. Histology shows a basal protein-secreting zone, enlarged secretory vesicles, lumina, drainage channels, and apparent exocytosis. The capsule is relatively thin, and the dentition is unspecialised rather than fang-like.	Transcriptomic evidence includes CRiSP, cystatin, kallikrein, lectin, B-type natriuretic peptide/helokinestatin, Kunitz, nerve growth factor, and group III phospholipase A2 homologues. Mandibular-gland proteomics in two species recovered CRiSP, kallikrein, natriuretic peptide, and group III phospholipase A2; several sequence lineages show active diversification.	An arboreal, non-varanoid anguimorph possesses translated members of a candidate toxin-related repertoire in anatomically active oral glands. This supplies an anatomical and molecular bridge between the highly elaborated helodermatid and varanoid systems, but expelled secretion, natural delivery, realistic dose, and a secretion-specific fitness benefit remain untested.
*Celestus* and *Gerrhonotus*	Mandibular glands contain serous or seromucous protein-secreting compartments, central lumina, and ducts draining towards the tooth row, consistent with a relatively simple ancestral Anguoidea condition. *Celestus* is now often placed in Diploglossidae, but its bridging position within Anguoidea is unchanged by that nomenclatural shift.	Oral-gland libraries recovered CRiSP, kallikrein, natriuretic/helokinestatin, and group III phospholipase A2 classes. Celestoxin is a lineage-specific peptide with hypotensive activity; *Gerrhonotus* and *Heloderma* yielded homologous venom hyaluronidase sequences, and pancreatic-type ribonuclease lineages show derived sequence change.	The combination of gland structure, shared precursor innovations, lineage-specific novelty, and pharmacology is compatible with inheritance and divergence of an ancestral oral toxin system. Protein-level secretion data, natural delivered dose, and ecological performance remain important gaps.
*Shinisaurus*	Mandibular glands are small and seromucous, with a low proportion of protein-secreting cells relative to other anguimorphs; the species also lacks grooved teeth. The oral gland is present and comparatively small and protein-poor, rather than an unexamined absence.	A recent mandibular-gland proteomic analysis detected no proteins that could be assigned to a venom repertoire. This is a targeted present-day negative result, not merely missing secretion data.	The result materially reduces support for current venom function and may support absence for the assayed secretion under the tested conditions. It cannot, by itself, distinguish primitive absence from secondary reduction or loss; that polarity requires explicit ancestral-state reconstruction.
*Heloderma*	Large, thickly encapsulated mandibular gland complex with extensive storage lumina, separated protein- and mucus-secreting regions, six principal compartments, ducts opening beside the lower teeth, and a bite-and-chew delivery behaviour. This is a highly elaborated system, not the inferred ancestral anatomy.	Venom proteomes contain kallikrein-related enzymes, exendins, helokinestatins, CRiSPs, phospholipase A2, and other components. Demonstrated actions include kinin release and hypotension, activation of coagulation Factors XII and VII, ion-channel binding and neuromuscular depression, pain, and other clinically evident effects.	The secure venom status of Heloderma establishes one specialised endpoint. Its shared molecular features with simpler anguimorph glands, together with the independent anatomy of varanoid glands, are consistent with elaboration from a broader ancestral secretory system.
*Varanus* and *Lanthanotus*	Independently enlarged and compartmentalised mandibular protein glands with thick capsules, enlarged storage lumina, and separation from mucus-secreting tissue. Ducts drain beside the mandibular teeth. Delivery remains low-pressure and bite-mediated; *Lanthanotus* lacks grooved teeth.	Proteomic and functional work recovers kallikreins and other toxin classes shared with anguids and *Heloderma*, alongside extensive lineage-specific variation. Representative *Varanus* venoms cause destructive fibrinogen cleavage, anticoagulation, cardiovascular effects, and ion-channel binding. *Lanthanotus* gland material is kallikrein-rich and releases kinins from kininogen in vitro.	A second origin of sophisticated gland architecture is clear, but a second origin of venom function is not required. The uncertain natural role of some varanoid secretions should be represented as graded or unknown evidence, rather than allowing anatomical simplicity or missing field data to decide the character state.

**Note:** ‘Gland extract’ proteomics confirms translated proteins within the gland but is not identical to analysis of expelled secretion or measurement of the dose delivered by a natural bite. The table therefore keeps anatomical, molecular, secretion, functional, and ecological claims distinct.

**Table 2:** Competing hypotheses and current evidentiary assessments.

Hypothesis	Core Prediction	Evidence Consistent with the Hypothesis	Main Unresolved Problem
Single shared functional origin	The relevant common ancestor already produced an oral secretion that entered bite wounds and increased fitness by perturbing recipient physiology. Later lineages changed dose, composition, gland position, and delivery efficiency, or reduced the system.	Distributed protein-secreting glands in both jaws; incomplete but directionally positive evidence in phylogenetically informative taxa within Anguimorpha and Serpentes; partly overlapping derived toxin lineages; coordinated expression across oral-gland locations; and abundant precedent for secondary reduction, loss, and replacement of venom function.	No study has yet demonstrated, across the lizard-snake divide, homology of the decisive toxin isoforms, secretory cell type, regulatory programme, secretion, and ecological function; current bridge evidence often lacks direct natural delivery, realistic dose, and secretion-specific fitness benefit in the same taxon. Shared ingredients and glands can predate venom function.
Shared precursor, two functional origins	The ancestor possessed homologous serous oral glands, regulatory capacity, and physiological proteins, but the anguimorph and snake lineages independently crossed the threshold to a wound-delivered secretion with an ecological toxic benefit.	A deeply conserved oral secretory network, broad non-oral expression of many venom-gene homologues, different later emphasis on mandibular versus maxillary glands, and independent recruitment within some protein families make repeated functionalisation plausible.	The functional threshold is difficult to reconstruct. Incomplete positive evidence near the bases of both focal lineages could reflect an earlier shared function, separate later gains, or false-positive classification; distinguishing two gains from inheritance followed by reduction requires fitted transition histories.
Two wholly independent origins	Lizard and snake venoms arose separately from ordinary oral tissues, and similarities reflect parallel recruitment from ancient physiological gene families rather than inheritance of a specialised oral substrate or toxic role.	The dominant proteomes and mature gland architectures differ, and many toxin-family homologues have widespread physiological expression. Several cross-lineage family matches do not resolve orthology or toxin status.	This model treats the distributed serous oral field, shared regulatory substrate, derived oral products, and retained low-output systems as parallel or nonvenomous outcomes rather than evidence of functional continuity. It is an explicit extreme alternative rather than the shared-precursor model fitted by Wüster, Modahl, and Arbuckle.
Current assessment: data deficient	Existing observations fit more than one model because the taxa, secreted proteomes, cell types, delivered doses, and ecological functions needed to discriminate them remain unsampled or incomparable.	The same distribution can be explained by inheritance with reduction or by repeated co-option from a shared substrate, and false-negative or false-positive tip classification can shift inference in opposite directions alongside differences in loss, hidden-state, and topology assumptions.	This review does not perform a comparative reconstruction and therefore does not rank the historical models. Resolution will require a hypothesis-driven comparative programme, especially in Iguania and early-diverging members of both focal lineages, once evidence-audited tip data are available.

**Note:** A ‘shared precursor’ is an anatomical and molecular substrate, not functional venom. It becomes venom only when a secretion is delivered through an inflicted wound and provides a fitness-relevant toxic effect. The current assessment is therefore data-deficient at the cross-lineage functional level, not at every subordinate anatomical or molecular level, and no relative ranking of the historical models is made here.

## Data Availability

No new data were created or analyzed in this study. Data sharing is not applicable to this article.
